# Transcriptome- Assisted Label-Free Quantitative Proteomics Analysis Reveals Novel Insights into *Piper nigrum*—*Phytophthora capsici* Phytopathosystem

**DOI:** 10.3389/fpls.2016.00785

**Published:** 2016-06-20

**Authors:** Chidambareswaren Mahadevan, Anu Krishnan, Gayathri G. Saraswathy, Arun Surendran, Abdul Jaleel, Manjula Sakuntala

**Affiliations:** ^1^Division of Plant Molecular Biology, Rajiv Gandhi Center for BiotechnologyThiruvananthapuram, India; ^2^Proteomics Core Facility, Rajiv Gandhi Center for BiotechnologyThiruvananthapuram, India

**Keywords:** plant-oomycete interaction, label-free proteomics, proteogenomics, black pepper—*Phytophthora capsici*, ReViGO, BLAST2GO, Protein Lynx Global SERVER, STRING 9.1

## Abstract

Black pepper (*Piper nigrum* L.), a tropical spice crop of global acclaim, is susceptible to *Phytophthora capsici*, an oomycete pathogen which causes the highly destructive foot rot disease. A systematic understanding of this phytopathosystem has not been possible owing to lack of genome or proteome information. In this study, we explain an integrated transcriptome-assisted label-free quantitative proteomics pipeline to study the basal immune components of black pepper when challenged with *P. capsici*. We report a global identification of 532 novel leaf proteins from black pepper, of which 518 proteins were functionally annotated using BLAST2GO tool. A label-free quantitation of the protein datasets revealed 194 proteins common to diseased and control protein datasets of which 22 proteins showed significant up-regulation and 134 showed significant down-regulation. Ninety-three proteins were identified exclusively on *P. capsici* infected leaf tissues and 245 were expressed only in mock (control) infected samples. In-depth analysis of our data gives novel insights into the regulatory pathways of black pepper which are compromised during the infection. Differential down-regulation was observed in a number of critical pathways like carbon fixation in photosynthetic organism, cyano-amino acid metabolism, fructose, and mannose metabolism, glutathione metabolism, and phenylpropanoid biosynthesis. The proteomics results were validated with real-time qRT-PCR analysis. We were also able to identify the complete coding sequences for all the proteins of which few selected genes were cloned and sequence characterized for further confirmation. Our study is the first report of a quantitative proteomics dataset in black pepper which provides convincing evidence on the effectiveness of a transcriptome-based label-free proteomics approach for elucidating the host response to biotic stress in a non-model spice crop like *P. nigrum*, for which genome information is unavailable. Our dataset will serve as a useful resource for future studies in this plant. Data are available via ProteomeXchange with identifier PXD003887.

## Introduction

Plants have developed a complex multi-layered molecular response against bacteria, fungi, and many oomycete pathogens. The plant immune system consist of at least two branches, the pathogen-associated molecular pattern (PAMP)-triggered immunity or PTI which directly recognizes small molecules from diverse microbes leading to early defense response and the effector-triggered immunity(ETI) which respond to specific molecules produced by the pathogen during their pathogenicity on the host plant (Jones and Dangl, 2006). Both branches of immunity involve several immune responses such as reactive oxygen species(ROS) production, an influx of Ca^2+^, activation of Mitogen activated protein kinases (MAPK), production, and regulation of phytohormones eventually culminating into transcriptional reprogramming.

*Piper nigrum* L., commonly referred to as black pepper, is a major non-model spice crop grown pantropically especially in southern peninsular India and Southeast Asia (Anandaraj and Sarma, 1995). The filamentous phytopathogen, *Phytophthora*, have devastating effect on a wide range of plants important to agriculture (Fisher et al., 2012). Two major pathogens of this groups, *Phytophthora infestans* and *Phytophthora capsici* produce late blight in potato and tomato crops, and they affect scores of crops leading to decrease in crop productivity and revenue loss (Lamour et al., 2012). The production of black pepper has significantly been affected by the hemibiotrophic oomycete, *P. capsici*. The pathogenicity of *P. capsici* toward black pepper causes foot or root rot disease and over the last century, it has significantly affected the production of peppercorn by local farmers. A systematic study of host immune responses and resistance mechanisms of black pepper against this pathogen has not been possible owing to lack of genome, transcriptome or proteome information of this plant (Gordo et al., 2012). The public databases reveal 206 nucleotide and 89 predicted protein sequences (22/02/2016) for this plant which suggest the deficiency in the available molecular data.

High-throughput -omics techniques like proteomics, transcriptomics or metabolomics have recently been widely adopted by plant biologist for studying the interaction of plants with other organisms (Mochida and Shinozaki, 2011). Recently, label-free quantitative proteomics methods merged with nano-LC-MS/MS have paved the way for comprehensive proteomic analysis in *Arabidopsis thaliana* (Niehl et al., 2013) and non-model plants like *Nicotiana attenuata* (Weinhold et al., 2015), and *Zingiber zerumbet* (Mahadevan et al., 2014). Several advantages that make the label-free method a high-throughput technique include gel-free handling of proteins, in-solution trypsin digestion and use of internal peptide standards leading to label-free quantification of protein abundance. This technique can be effectively used for identification of novel proteins from non-model organisms for which genome information is very limited or totally lacking.

In our present study, we report a transcriptome assisted nanoUPLC-MS^E^ based label-free quantitative proteomics approach in *P. nigrum* where we have efficiently used a gel-free proteomics approach combined with a novel identification approach using a black pepper leaf transcriptome to obtain proteomic information from host leaves when challenged with *P. capsici*. We have attempted to understand the proteome changes associated with immune responses of black pepper against *P. capsici* infection at 24 h post inoculation. This study is the first to identify, characterize, and quantitate novel proteomics information in black pepper as well as produce an expression data on this important phytopathosystem. Our simple and integrative approach to derive and understand the molecular changes involved in plant-oomycete interaction is pivotal for the advancement of plant immunity research. Our study brings novel insights specifically involved in black pepper—*Phytophthora* interaction which is of practical interest for developing crop improvement strategy in this plant.

## Materials and methods

### Plant material

For our present study, we used 8–12 weeks old black pepper (*P. nigrum* L. variety - Panniyur I) plants, grown, and maintained in a greenhouse at Rajiv Gandhi Center for Biotechnology, Thiruvananthapuram, India. *P. capsici* virulent to black pepper was obtained from Kerala Agricultural University, Thiruvananthapuram, India. We followed *P. capsici* mycelial agar plug inoculation method (Krishnan et al., 2015) on detached leaves described by Zuluaga and co-workers (Zuluaga et al., 2015) for infection assay experiment. *P. capsici* was axenically grown on potato dextrose agar medium (PDA) at 28°C for 4 days prior to inoculation. The abaxial side of the second and third leaf detached from the top of at least 10 different plants were pinpricked once and inoculated with *P. capsici* mycelial agar plug or sterile PDA plugs (mock control) at multiple sites. The leaves were placed upside-down in humid transparent plastic trays and incubated for 24 h in a controlled growth chamber (Conviron, Canada) at 24°C and a photoperiod of 16 h and an RH of 70% prior to sampling. After incubation, the agar plugs from the site of infection were manually removed. Leaf discs of 7.0 mm diameter were harvested using a paper hole puncher from the inoculation site of all the leaves and immediately frozen in liquid nitrogen (Zuluaga et al., 2015) prior to total RNA and protein isolation. We inoculated at least 4-6 different areas on a detached leaf. Sampling was done by combining 40–50 leaf discs per set were pooled for each experimental condition.

### Trypan blue staining of *P. capsici*

For microscopic observation of *P. capsici*, we followed an established trypan blue staining technique (Chung et al., 2010). In short, the leaf discs were submerged in solution A (acetic acid: methanol, 1:3 v/v) for 10–12 h. After the initial incubation, solution A was replaced by solution B (acetic acid: ethanol: glycerol, 1:5:1 v/v/v) and tissues were incubated for 3–6 h. Later, solution B was removed and replaced by a staining solution which includes 0.01% trypan blue in lactophenol and the leaf discs were incubated for 6–12 h. Finally, the staining solution was removed and the leaf discs were rinsed with 60% glycerol twice. Leaf discs were mounted on slides with 60% glycerol and observed under 40X magnification under a compound microscope (Leica Microsystems DM750).

### Protein isolation, solubilization, and filter-aided sample preparation (FASP) of protein for label-free mass spectrometry

Total protein was extracted from *P. capsici* infected and mock control leaves separately as described earlier (Isaacson et al., 2006). For solubilization of protein, the lyophilized protein pellet was vortexed at room temperature for 1 h in 10 mL of 7 M urea and 2 M thiourea and 1 mM PMSF. The resolubilized protein was centrifuged at 14,000 g for 15 min in a refrigerated centrifuge (Eppendorf, 5810R, Germany). For the preparation of proteins suitable for mass spectrometry, we improvised an FASP technique (Wiśniewski et al., 2011) where a sequential buffer exchange step using 3KDa spin filtration units (Millipore) was followed using 50 mM ammonium bicarbonate (Sigma) as exchange buffer. Briefly, 200 μl of the clear supernatant was mixed with 200 μl of 50 mM ammonium bicarbonate, loaded on to pre-activated Amicon ultra 0.5 mL centrifugal filters (Millipore) and centrifuged at 14,000g at 20°C for 45 min. The addition of exchange buffer was repeated twice for complete removal of salts from the solubilized protein. The solubilized protein sets were then quantified using Bradford method (Bradford, 1976).

### Trypsin digestion, liquid chromatography, and MS analysis

For preparation of tryptic peptides, an aliquot equivalent to 100 μg of the concentrated protein was taken separately for each sample. The samples were normalized to a final concentration of 1 μg/μl using 0.1% (w/v) of Rapigest SF (Waters, Milford, MA) prepared in 50 mM ammonium bicarbonate (Sigma). Preparation of tryptic peptides was carried out following the protocol described earlier (Gopinath et al., 2015). Briefly, protein disulfide bonds were reduced by treating the sample with 5 μL of 100 mM DL-dithiothreitol in 50 mM ammonium bicarbonate for 30 min at 60°C and alkylated with 200 mM iodoacetamide in 50 mM ammonium bicarbonate at room temperature for 30 min in the dark. Proteins were then digested by using trypsin (Sequence grade, Sigma) modified in 50 mM ammonium bicarbonate by incubating overnight at 37°C. The trypsin digestion reaction was stopped by adding 1 μL of 100% formic acid. The solutions containing digested peptides were centrifuged at 14000 rpm for 12 min, and the collected supernatant was stored at −20°C until LC/MS/MS analysis.

For protein profiling and label-free quantitative proteomic analysis, we spiked the peptide samples with appropriate peptide standards following previous report (Mahadevan et al., 2014) optimized for label-free protein quantitation. Briefly, appropriate peptide standards of various known proteins were spiked in different ratios among the samples using Mass PREP Digestion Standard (MPDS, Waters) which is a set of tryptic digested peptides from four proteins, namely, Bovine Serum Albumin (BSA), Rabbit Glycogen Phosphorylase b (GPB), Yeast Alcohol Dehydrogenase (ADH), and Yeast Enolase I (ENO). These MPDS (Waters) were available as Protein Expression Mixture 1(MPDS 1) and Protein Expression Mixture 2 (MPDS 2) having 1:2:8:0.5 fold abundance of peptides for ADH, ENO, BSA, and GPB, respectively, in MPDS 2. MPDS 1 was spiked in the control sample and MPDS 2 in the test sample for eventual protein expression analysis using a label-free method.

The nanoUPLC-MS^E^ acquisition of tryptic peptides was carried out according to Gopinath et al. (2015). Briefly, the tryptic peptides were separated using a nanoACQUITY UPLC® chromatographic system (Waters, Manchester, UK) were the peptides were separated by reversed-phase chromatography technology. Instrument control and data processing was taken care with the help of MassLynx4.1 SCN781 software. The peptide sample was injected in partial loop mode in 5 μl loop (final injection volume was 3.0 μl which contains ~3 micrograms of tryptic digested peptides). Water was used as solvent A and acetonitrile was used as solvent B. All solvents for the UPLC system contained 0.1% formic acid. The tryptic peptides were trapped and desalted on a trap column (Symmetry® 180 μm × 20 mm C18 5 μm, Waters) for 1 min at a flow rate of 15 μl/min. The trap column was placed in line with the reversed-phase analytical column, a 75 μm i.d. X 200 mm BEH C18 (Waters) with particle size of 1.7 μm.Peptides were eluted from the analytical column with a linear gradient of 1 to 40% solvent B over 55.5 min at a flow rate of 300 nl/min followed by a 7.5 min rinse of 80% solvent B. The column was immediately re-equilibrated at initial conditions (1% solvent B) for 20 min. The column temperature was maintained at 40°C. The *lock mass*, [Glu^1^]-Fibrinopeptide B human (Sigma) (positive ion mode [M+2H]^2+^ = 785.8426) for mass correction was delivered from the auxiliary pump of the UPLC system through the reference sprayer of the NanoLockSpray™ source at a flow rate of 500 nl/min. Each sample was injected in triplicate with blank injections between each sample.

Mass spectral analysis of eluting peptides from the nanoACQUITY UPLC® was carried out on a SYNAPT® G2 High Definition MS™ System (HDMS^E^ System, Waters). The instrument settings were: nano-ESI capillary voltage–3.3 KV, sample cone–35 V, extraction cone–4 V, IMS gas (N_2_) flow–90 (mL/min). To perform the mobility separation, the IMS T-Wave™ pulse height is set to 40 V during transmission and the IMS T-Wave™ velocity was set to 800 m/s. The traveling wave height was ramped over 100% of the IMS cycle between 8 V and 20 V. All analyses were performed using positive mode ESI using a NanoLockSpray™ source. The lock mass channel was sampled every 45 s. The time of flight analyzer (TOF) of the mass spectrometer was calibrated with a solution of 500 fmole/μL of [Glu^1^]-Fibrinopeptide B human (Sigma). This calibration set the analyzer to detect ions in the range of 50–2000 *m/z*. The mass spectrometer was operated in resolution mode (V mode) with a resolving power of 18,000 FWHM and the data acquisition was done in *continuum* format. The data was acquired by rapidly alternating between two functions—Function-1 (low energy) and Function-2 (high energy). In Function-1, we acquire only low energy mass spectra (MS) and in Function-2, we acquire mass spectra at elevated collision energy with ion mobility (HDMS^E^. In Function-2, collision energy was set to 4 eV in the Trap region of mass spectrometer and is ramped from 20 to 45 eV in the Transfer region of mass spectrometer to attain fragmentation in the HDMS^E^ mode. The *continuum* spectral acquisition time in each function was 0.9 s with an interscan delay of 0.024 s.

### Data processing, protein identification, and label-free quantitation

The acquired ion mobility enhanced MS^E^ spectra was analyzed using Protein Lynx Global SERVER™ v2.5.3 (PLGS, Waters) for protein identification and label-free relative protein quantification. Data processing included *lock mass* correction post acquisition. Processing parameters for PLGS were set as follows: noise reduction thresholds for low energy scan ion—150 counts, high energy scan ion—50 counts and peptide intensity—500 counts (as recommended by the manufacturer). The default setting in PLGS (“automatic”) was used for the precursor ion and fragment ion mass tolerance in which the mass tolerance depends on the resolution of the mass spectrometer. For protein identification and quantification, the obtained raw data were searched against a target-decoy black pepper protein database created using a publically available transcriptome dataset for black pepper leaf (Joy et al., 2013). Briefly, raw reads were obtained from NCBI SRA accession number SRX119532. A transcriptome database was constructed as described by Evans et al. (2012), where Trinity (vr2012-10-05) (Grabherr et al., 2011) *de novo* assembly software was used to generate an output of assembled transcripts using default parameters. A total of 169165 entries were generated with contig N50 of 1188 bp. The Trinity-generated transcripts were used to predict and create a custom protein database with open reading frames (>200 nucleotides) from all six frames using the EMBOSS tool getorf (Rice et al., 2000) provided on the Galaxy-P website (https://usegalaxyp.org). The parameters opted include a standard translation with start codon of methionine (M), translation was fixed between start and stop codons, number of flanking nucleotides were kept at 100 bp and the ORF prediction were allowed in the reverse complement as well. The protein identifications were obtained by searching against this custom generated black pepper database. During database search, the protein *false positive rate* was set to 4%. The parameters for protein identification were followed as described earlier (Gopinath et al., 2015). The identification of a peptide required, at least, one fragment ion match, detection of a protein required at least three fragment ion matches and a protein was required to have at least one peptide match for identification. Variable modification and fixed modification were selected as oxidation of methionine and cysteine carbamidomethylation respectively. The enzyme was chosen as trypsin with a specificity of one missed cleavage.

For label-free protein quantification, datasets were normalized using the PLGS “auto-normalization” function. All protein hits were identified with a confidence of >95%. Yeast Alcohol dehydrogenase (ADH) was used as the internal standard for normalization during relative quantification. Quantitative analysis was performed by comparing the normalized peak area/intensity of identified peptides between the samples. In principle, any non-changing peptide ion can be regarded as a spike peptide along with the internal spike peptides already added during sample peptide preparation. Proteins identified in at least two of the three injections were only considered for expression analysis. For label-free quantitation, the expression fold changes were fixed as described (Meng et al., 2011) where only those proteins with a ratio of either ≥1.2 or ≤ 0.80 (±0.20 natural log scale) were regarded as significantly up- or down-regulated respectively. Concurrently, we obtained parameters such as score, sequence coverage and the number of peptides identified for each protein which is representative of the expression analysis of samples.

The mass spectrometry proteomics data have been deposited to the ProteomeXchange Consortium via the PRIDE (Vizcaíno et al., 2016) partner repository with the dataset identifier PXD003887 and 10.6019/PXD003887.

### Functional annotation and bioinformatics analysis

All the protein sequences corresponding to protein hits identified by MS/MS analysis were manually retrieved in FASTA format from the custom black pepper protein database. The protein sequences were also separated in batches namely—differentially expressed proteins, test proteins (proteins present only in *P. capsici* infected leaf protein dataset) and control proteins (proteins present only in mock control leaf protein dataset). The retrieved sequences were loaded on Blast2Go 3.0 software (http://www.blast2go.com) (Conesa et al., 2005; Götz et al., 2008) which is a comprehensive high-throughput tool for gene ontology (GO) (Ashburner et al., 2000) online tool. ReViGO removes the redundant terms, calculates and summarizes the list of GO terms according to the enrichment in the cellular component, biological process, and molecular function and helps visualization of the remaining GO terms based on their semantic similarities in scatterplots. The differentially expressed proteins were also checked for their enrichment in KOBAS 2.0 software (http://kobas.cbi.pku.edu.cn/) (Xie et al., 2011). Protein–protein interaction for the differentially regulated protein dataset was derived from the STRING (Search Tool for the Retrieval of Interacting Genes/Proteins) database version 9.1 (www.stringdb-org) (Franceschini et al., 2013). Differentially down-regulated protein sequences derived from our quantitative proteomics analysis were manually retrieved and were mapped to STRING protein database of *A. thaliana*. Only those protein interactions with a high confidence score (≥0.9) were retained in the network. Further, TargetP1.1 (http://www.cbs.dtu.dk/services/) (Emanuelsson et al., 2007) was used to predict sub-cellular localization of all the proteins.

### RNA isolation, cDNA synthesis

Total RNA was extracted from pooled sets of *P. capsici* infected and mock control leaf discs separately using RNeasy plant mini kit (Qiagen) following manufacturers' instructions. Prior to cDNA synthesis, all samples were equally treated with DNase I (Ambion, Austin, TX).First-strand cDNA synthesis was carried out using 2 μg of total RNA from each sample along with oligo (dT) 15-mer primers (Promega, Madison, WI), 200 units of MMLV reverse transcriptase (Promega, Madison, WI), and 40 units of ribonuclease inhibitor RNasin (Promega, Madison, WI).

### Candidate gene identification, quantitative reverse transcription PCR (qRT-PCR), cloning, and sequence characterization

The full-length nucleotide sequences corresponding to the protein hits identified by MS/MS analysis were predicted from the custom black pepper protein database using Geneious R8 software and were retrieved manually in FASTA format. All predicted transcripts were confirmed by their orientation with respect to their start/stop codon. Primer sequences for all the selected genes for qRT-PCR and full-length cloning were designed with the Primer3Plus tool (http://primer3plus.com/cgi-bin/dev/primer3plus.cgi) (Untergasser et al., 2007) available with Geneious R8 software. The amplicons were further cloned into pGEM-T-easy Vector system (Promega) and transformed in *E. coli* DH5α (Invitrogen) according to the manufacturer's recommendations. The plasmid DNA was isolated from the positive *E. coli* cells, digested with EcoRI for validation of their insert size and the inserted DNA were sequenced in both directions using the BigDye Terminator method on an auto-sequencer (Applied Biosystems) using vector specific primers.

For qRT-PCR analysis, we followed SYBR Green I technology (Applied Biosystems) using Power SYBR Green PCR Master Mix (Applied Biosystems) on ABI 7900 Real-Time PCR System (Applied Biosystems, Foster City, USA). The qRT-PCR conditions were followed as described earlier (Asha et al., 2012). The housekeeping gene *P. nigrum18srRNA* was used for normalization of expression data. Primer dimers and multiple product formations were ruled out using melting-curve analysis immediately after the PCR. The fold changes of selected genes were calculated as log_10_ relative quantity (RQ) values using the comparative C_T_ (2^−ΔΔCt^) method (Schmittgen and Livak, 2008). All the data generated will be made available to all interested researchers upon request.

## Results and discussion

### Black pepper shows early susceptibility to *P. capsici*

**Figure 1:**
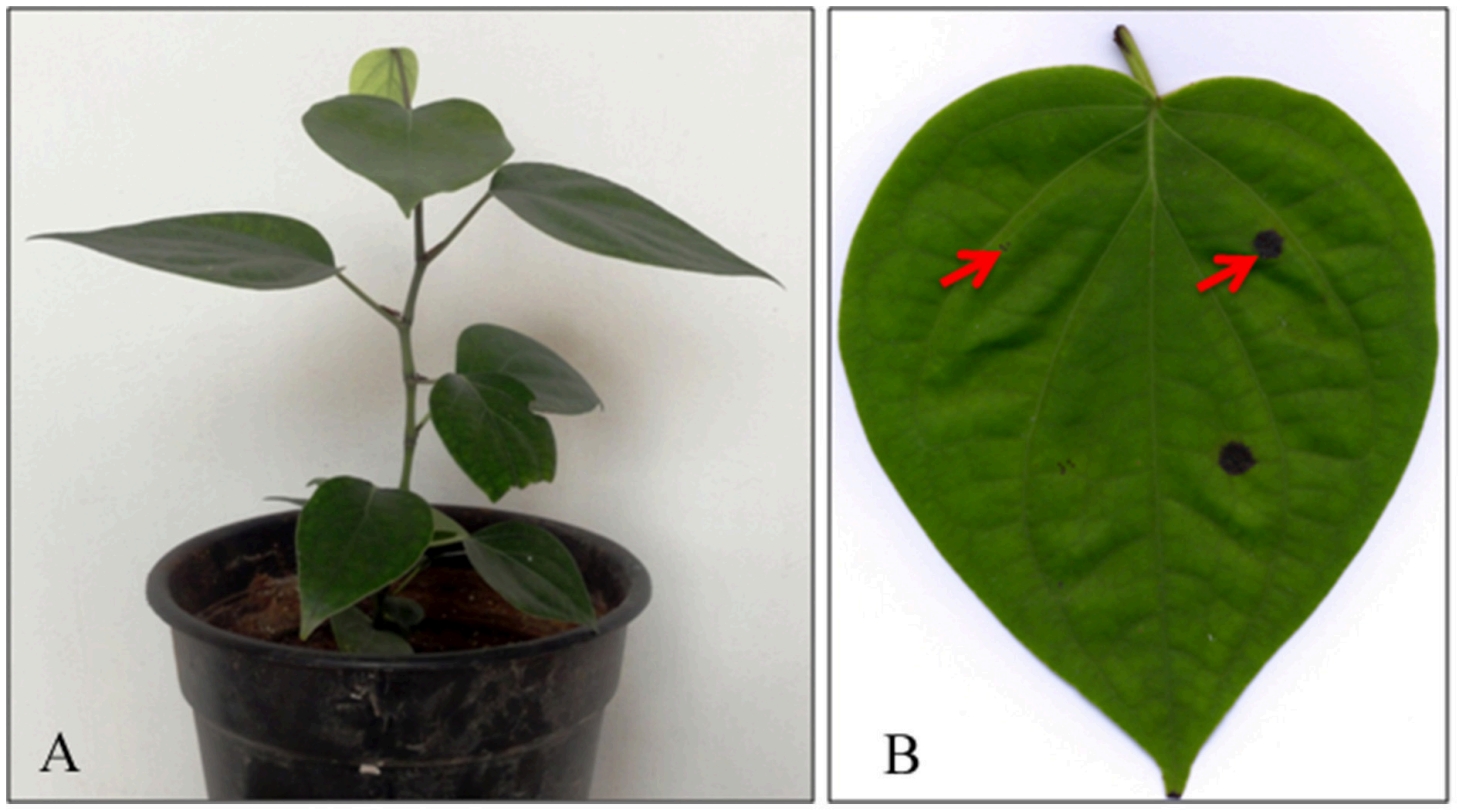
**(A)** Young plant of *Piper nigrum* L. and **(B)** Macroscopic evaluation of symptoms caused by *Phytophthora capsici* on detached leaves. Symptoms were monitored at 24 hpi were the pathogen produces water soaked lesion at the site of infection. The red arrow denotes examples of the 7-mm diameter leaf disc areas from which total RNA and protein were extracted for the study.

**Figure 2:**
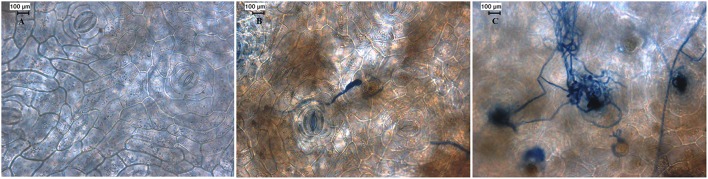
**Microscopic assessment of black pepper leaves challenged with *P*. *capsici*. (A)** 0 hpi, **(B)** 12 hpi, and **(C)** 24 hpi. The detached leaf disc was prepared for trypan staining followed by microscopy. Scale bar: **(A–C)** 100 μm.

We conducted preliminary experiments to demonstrate the interaction between black pepper (*P. nigrum* L. v Panniyur 1; Figure 1A) with *P. capsici* in a time-course experiment. Detached leaves of black pepper were inoculated with either mycelial plugs of *P. capsici* grown on PDA medium or sterile PDA plugs (Figure 1B). Macroscopic observations of the infection site were carried out at 12 h interval through 0 to 24 hpi. Multiple inoculations showed that the initial progression of *P. capsici* was observed within 12 hpi when slight browning leading to water-soaked lesions were observed (Figure 2B). A similar mode of pathogenicity have been observed on *P. parasitica*–tomato (Le Berre et al., 2008) and *P. capsici*–*A. thaliana* (Wang et al., 2013) pathosystems. At 24 hpi, distinct phenotypic changes suggesting the complete susceptibility of black pepper leaf tissue to *P. capsici* were observed which included extensive browning of the inoculation site with water-soaking, tissue collapse, and cell death (Figure 1B). Based on these observations, microscopic observation of the infection was carried out using trypan blue staining of *P. capsici* infected leaf discs *in planta* along with mock control (Figure 2A). Microscopically, the inoculation site of the infected leaves showed progressive developmental stages of *P. capsici*, like germination of oospores, mycelial growth, and formation of haustorial structures (Figure 2B). *P. capsici* seems to enter the leaf tissue both through the epidermal cell walls and were observed penetrating via stomatal cavities. Within 24 hpi, widespread colonization of *P. capsici* in and around the stomatal region was observed (Figure 2C). The mycelial growth spreads radially toward other healthier leaf sectors as evident from our experiment. Our observation also suggests that being a hemibiotroph, *P. capsici* might have a fast reproductive cycle during its infection on black pepper, and may involve an early transition from biotrophy to necrotrophy within 24 hpi. Similar observation have been reported in *P. capsici*–tomato pathosystem (Jupe et al., 2013). Considering the early hemibiotrophic transition of *P. capsici* on black pepper, we presume that the host proteome expression, its biological regulation toward defense response against the pathogen and its repertoire of resistance genes could significantly be altered. In order to get a coordinated insight into the host response following the oomycete infection, we have tried to understand the expression analysis of the leaf proteome of black pepper challenged with *P. capsici* at 24 hpi.

### Label-free proteomics approach to study the black pepper—*P. capsici* interaction

To understand the proteome changes underlying disease progression in black pepper, we initiated a high-throughput label-free quantitative proteomics study comparing mock infected control leaf sets and leaves challenged with *P. capsici*. For the precise identification of the host proteins, we utilized a transcriptome assisted proteomics strategy. One such rare study which used a transcriptomics assisted proteomic analysis approach was reported in *Nicotiana occidentalis* infected by *Candidatus* Phytoplasma mali strain AT (Luge et al., 2014) where the author used a leaf transcriptome dataset and further coupled it with a dimethyl labeling approach for quantitative MS analysis to reveal novel metabolic pathways which were affected in *N. occidentalis* during Phytoplasma infection. It is already accepted that publically available dataset could be utilized for generating custom protein database (Evans et al., 2012). In *P. nigrum*, we could identify only two high-throughput transcriptome profiling datasets (Gordo et al., 2012; Joy et al., 2013) but almost no sequence and protein expression data are available till now. Hence in our study, we used the black pepper leaf transcriptome dataset (Joy et al., 2013) publically available with NCBI SRA accession number SRX119532 for constructing a black pepper protein database. The methodology followed for creating the target protein database has been discussed earlier (Evans et al., 2012) in the methods section. Our custom protein database generated 231,777 predicted protein sequences in all six frames covering the 169165 transcript sequences generated by *de novo* assembly of the transcriptome.

Label-free quantitative proteomics has recently evolved as a high-throughput technique for identification and quantitation of proteins from diverse plants like cotton (Meng et al., 2011), *Arabidopsis* (Niehl et al., 2013), *Z. zerumbet* (Mahadevan et al., 2014), and peanut (Kottapalli et al., 2013). Here we have utilized an improved protein sample preparation strategy, along with an in-solution protein digestion followed by a nanoUPLC-MS^E^ method for identification of tryptic peptides generated from the spiked protein samples. Ion mobility enhanced MS^E^ spectra were derived from three technical runs and were completely analyzed using Protein Lynx Global SERVER v2.5.3 (PLGS, Waters) for protein identification and label-free quantitative proteomics analysis. The *continuum* nLC-MS/MS analyzed raw data of all the fractions of mock control and *P. capsici* infected samples were processed employing the transcriptome derived black pepper protein database and then merged as single *control* file (mock control) and *test* file (*P. capsici* infected) respectively. The label-free proteome profile identified 151,189 peptides of which 5870 were unique peptides which eventually corresponded to 532 novel protein hits (Table S1). More than 95% of the proteins were identified with at least two uniquely matched peptides (Table S1). A wide range of proteins with individual PLGS score of well over 20 (Yerlikaya et al., 2015) was identified in the present study for which the average molecular weight predicted by PLGS (Table S1) varied from163564Da (comp88403_c0_seq2_4) to 7068Da (comp79043_c0_seq2_2).

### Identification of differentially expressed proteins using a label-free quantitative proteomics

**Figure 3:**
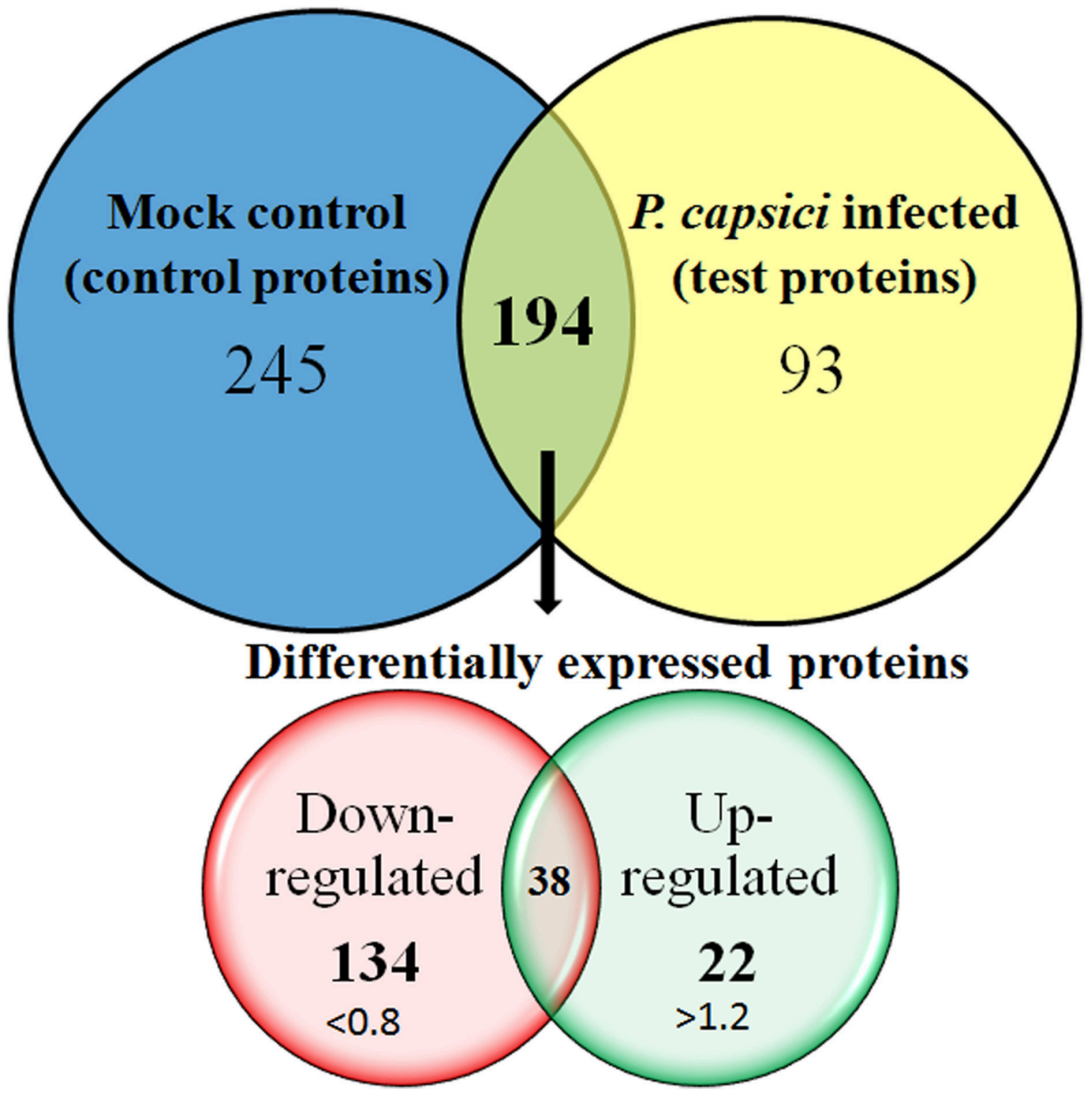
**Venn diagram representing the number of proteins identified in the leaf proteome of *Piper nigrum* in response to *Phytophthora capsici* infection**. Of 532 leaf proteins identified, 194 were grouped as differentially expressed proteins, 245 proteins were identified only on mock infected set and 93 were identified only on *P. capsici* infected set. Label-free proteome quantitation was carried out between infected and uninfected leaf samples challenged with or without *P. capsici* at 24 hpi.

The relationship between *Phytophthora* species with other hosts have extensively been studied, most of them based on transcript levels. Studies on *P. capsici*–tomato (Jupe et al., 2013), *P. sojae*–soybean (Moy et al., 2004), or *P. infestans* infecting potato (Ali et al., 2014) suggest that there occurs a significant change in the gene expression levels in the host plant. In the present study, we observe significant changes in the abundance level of 194 proteins out of 532 proteins. These were identified in both test and control dataset and were considered as differentially expressed proteins. The protein ratio for each of these proteins ranges from 0.10 for subtilisin-like protease (comp87657_c0_seq1_4) to a ratio of 5.87 which was identified for photosystem I reaction center subunit vi (comp65483_c0_seq1_1). The abundance level of all the other proteins was between these two ranges and was highly significant with *P*-values assigned by PLGS tool. Here, the significance was tested based on “*P*-value” calculations made by PLGS “auto-normalization” parameters and are considered as an advanced statistical test where the value between 0.95 and 1 indicates a 95% likelihood of up-regulation and a value between 0 and 0.05 represent a 95% likelihood of down-regulation. We identified 245 proteins uniquely present in control dataset and 93 in test protein dataset (Figure 3). For label-free quantitation, the protein ratio level of >1.2 (±0.20 natural log scale) was specified as a threshold to evaluate significantly up- or down-regulated proteins as described earlier (Meng et al., 2011; Mahadevan et al., 2014). We identified 134 significantly down-regulated proteins with a fold expression ratio ≤ 0.8 and at least 22 proteins were found to have fold expression ratio of ≥1.2 which corresponds to significantly up-regulated proteins (Figure 3).

### Functional annotation and classification of novel black pepper proteins

**Figure 4:**
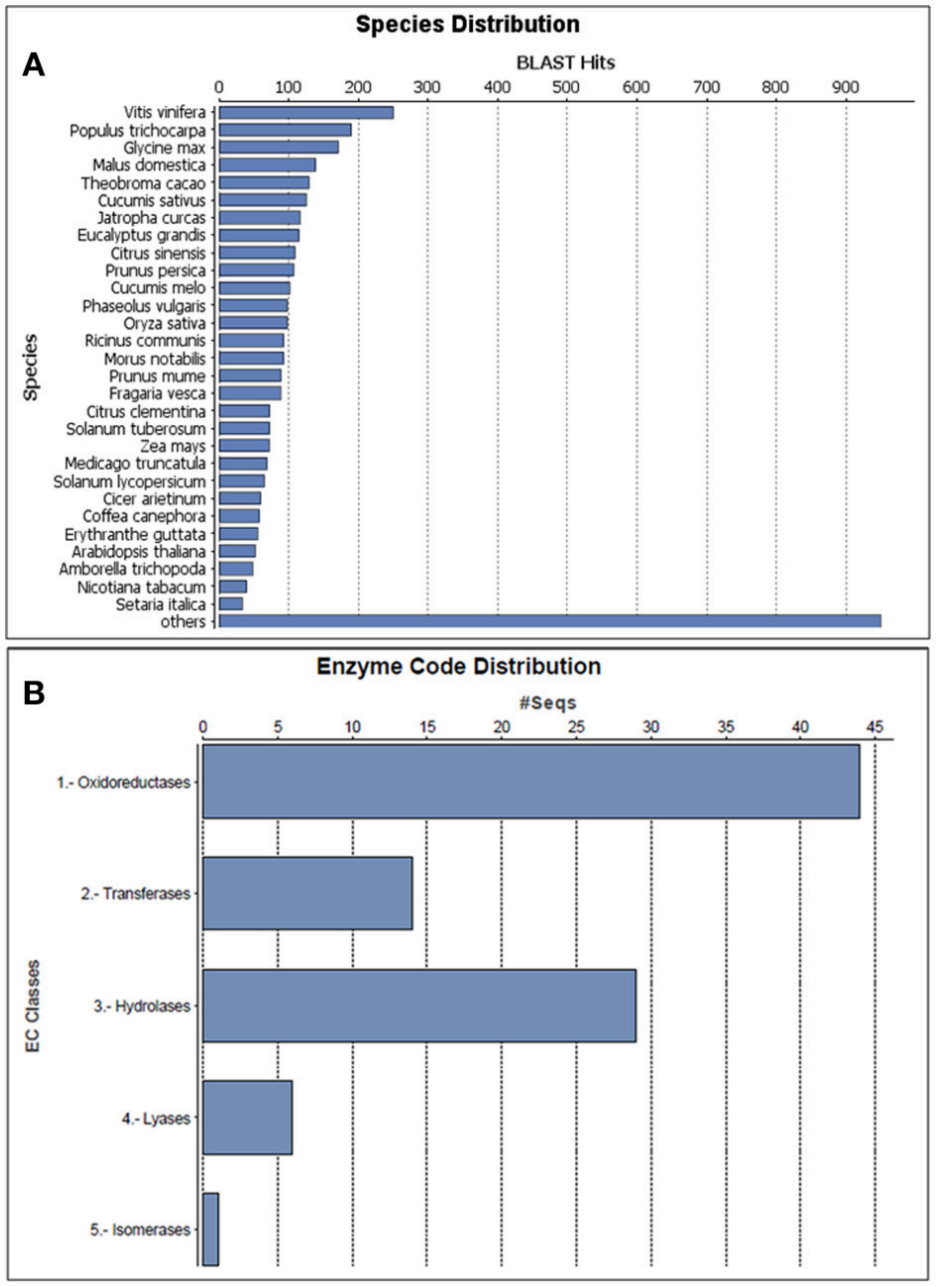
**Characterization of the proteins identified in black pepper based on a Viridiplantae protein database search. (A)** Species distribution of the top BLAST hits for the proteins predicted with a cutoff of *E* < 10^−3^ and **(B)** Enzyme code distribution of the proteins identified.

All the 532 protein sequences corresponding to their identified sequence name were manually retrieved for further functional annotation and bioinformatics analysis. A comprehensive functional annotation carried out using Blast2GO3.0 revealed novel leaf proteome of black pepper. The protein sequences were searched against *Viridiplantae* database and *A. thaliana* TAIR 10 protein database using NCBI BLASTP available with Blast2GO tool. In total, complete protein description and gene ontology were available for 518 proteins and have been represented and explained further. The homology-driven search of proteins showed similarity to a number of plant species, the single largest similarity to any species with respect to BLAST hits being *Vitis vinifera* followed by *Populus trichocarpa* and *Glycine max* (Figure 4A) to name a few. The enzyme code distribution of the identified proteins showed the largest representation of oxidoreductases followed by hydrolases and transferases (Figure 4B) which indicate the over involvement of plant defense components (Balmer et al., 2015). Our results are also in consensus with the earlier observations where the enzymatic activity and *in planta* localization of hydrolases and oxidoreductases were found to be higher during the interaction of *Zantedeschia aethipica* with *Pectobacterium carotovorum* (Luzzatto-Knaan et al., 2014).

The functional annotation gave a precise description for all the proteins and paved way for identification of novel proteins which are critical to several biochemical pathways and immune responses in black pepper (Tables S2A–C). All the proteins were also assigned with their enzyme commission (EC) numbers connecting them to Kyoto Encyclopedia of Genes and Genomes (KEGG) pathways where ever possible (Tables S3A–C). In our study, proteins were found to be involved in at least 49 biochemical pathways which potentially correlates with the plant defense response of black pepper resulting from the *Phytophthora* infection. The majority of the proteins were involved in pathways such as carbon fixation in photosynthetic organisms, glyoxylate and dicarboxylate metabolism, glycolysis/gluconeogenesis pathways, methane metabolism, cysteine and methionine metabolism, glycine, serine, and threonine metabolism, phenylpropanoid biosynthesis, pentose phosphate pathway, fructose and mannose metabolism, purine metabolism and glutathione metabolism (Table S3D).

**Figure 5:**
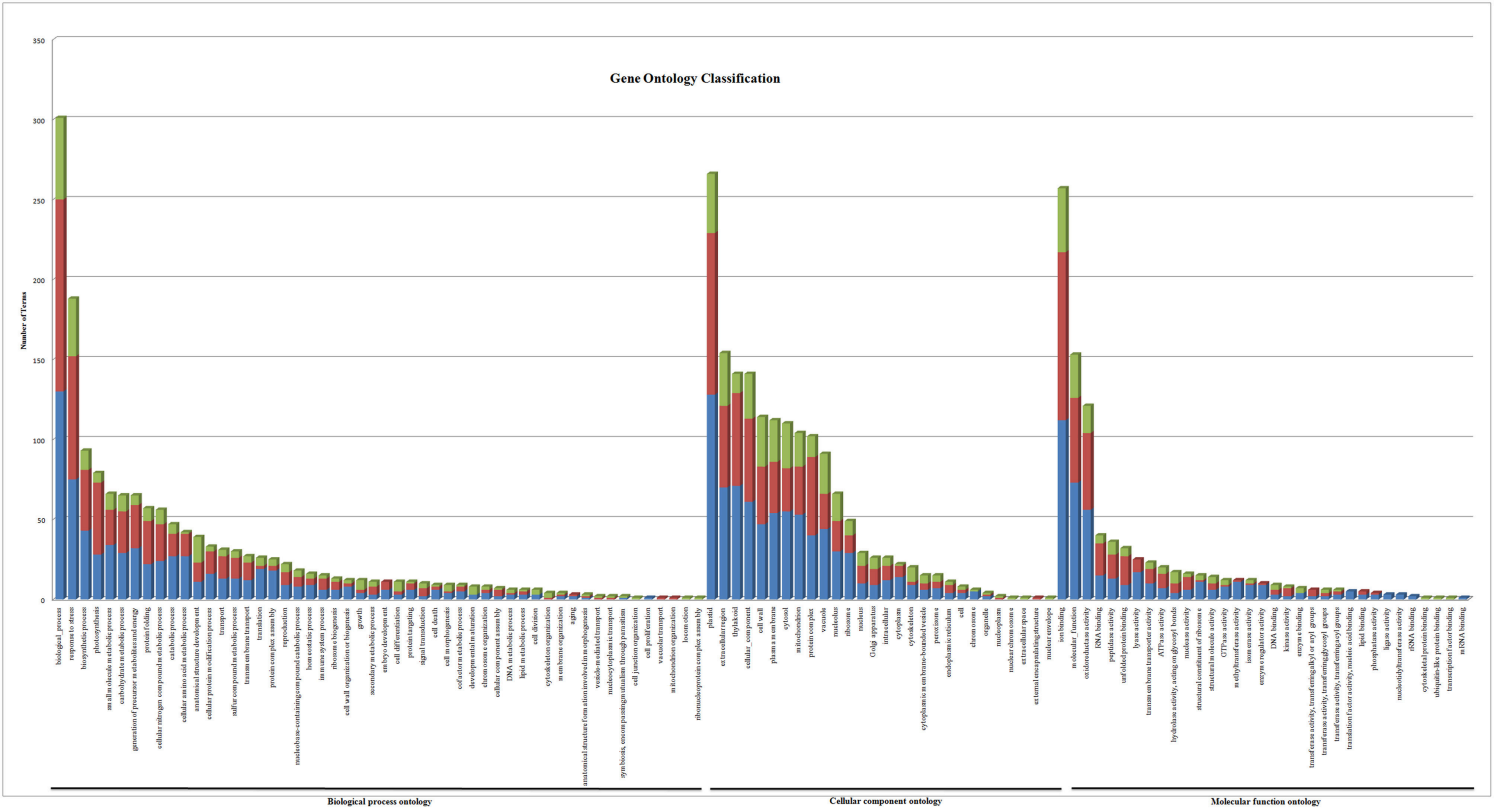
**Gene ontology classification of all the 532 proteins identified in black pepper**. The results are summarized in terms of three functional categories: cellular component, molecular function, and biological process. The blue bar represents proteins identified only in mock-infected protein set, the red bar represents GO terms for differentially expressed protein set and the green bar represents the proteins identified only in *P. capsici* infected dataset.

**Figure 6:**
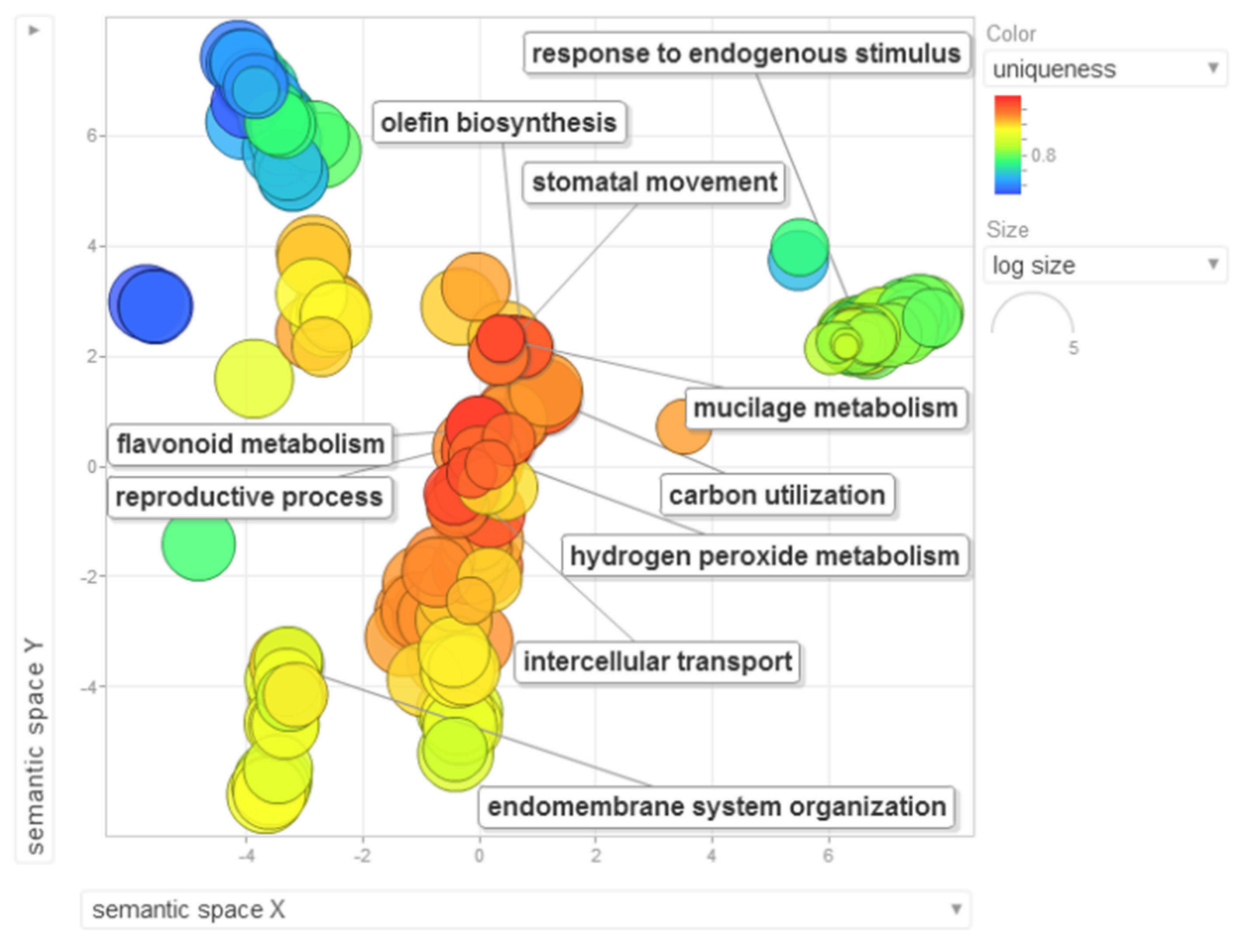
**The scatterplot visualizes the biological process terms identified for the 532 proteins of black pepper**. The bubble color indicates the uniqueness of the GO terms (the legends for the first 20 terms have been included in the figure). A detailed list of GO terms along with their uniqueness has been supplied as a Table S5A.

**Figure 7:**
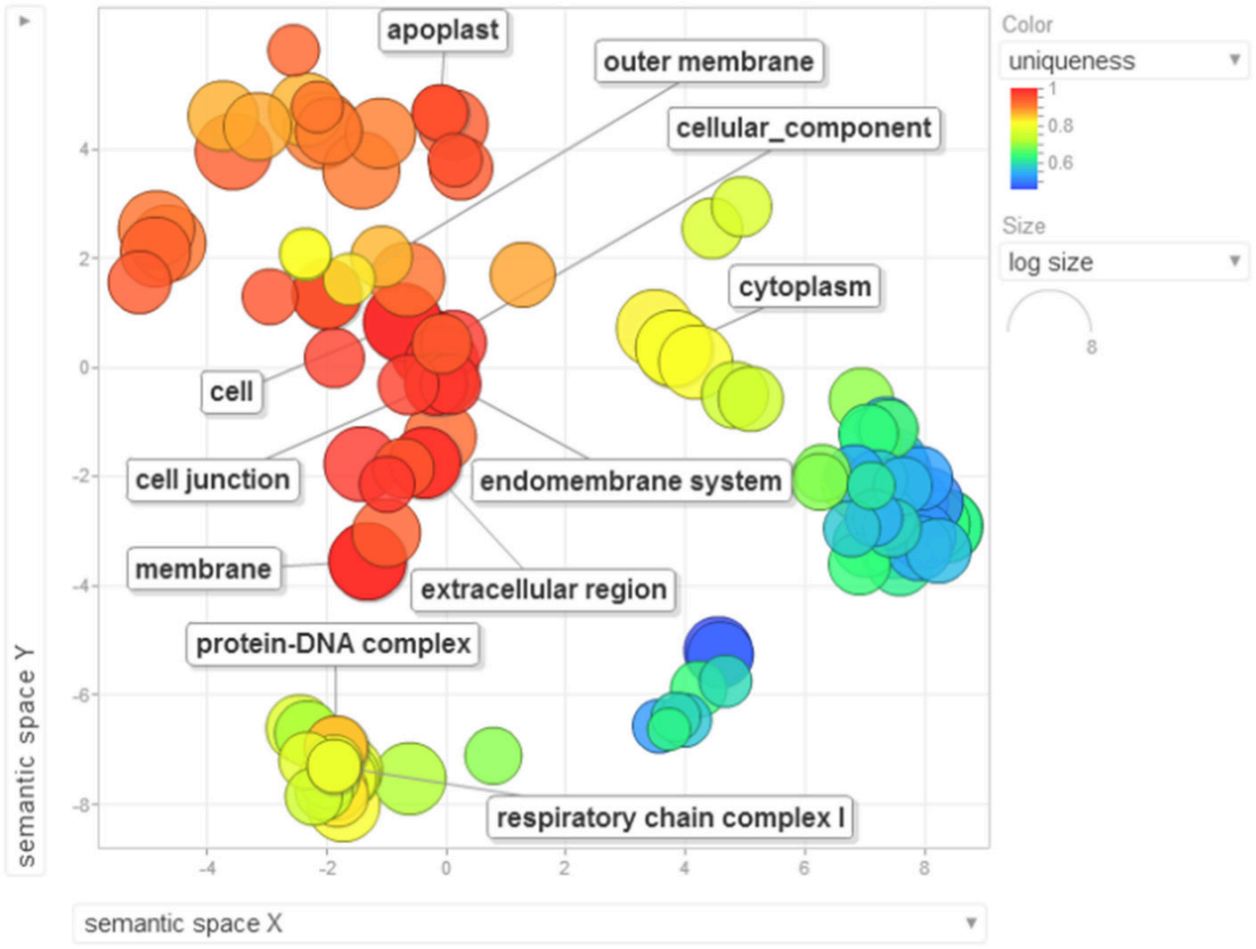
**The scatterplot visualizes the cellular component terms identified for the 532 proteins of black pepper**. The bubble color indicates the uniqueness of the GO terms (the legends for the first 20 terms have been included in the figure). A detailed list of GO terms along with their uniqueness has been supplied as a Table S5B.

**Figure 8:**
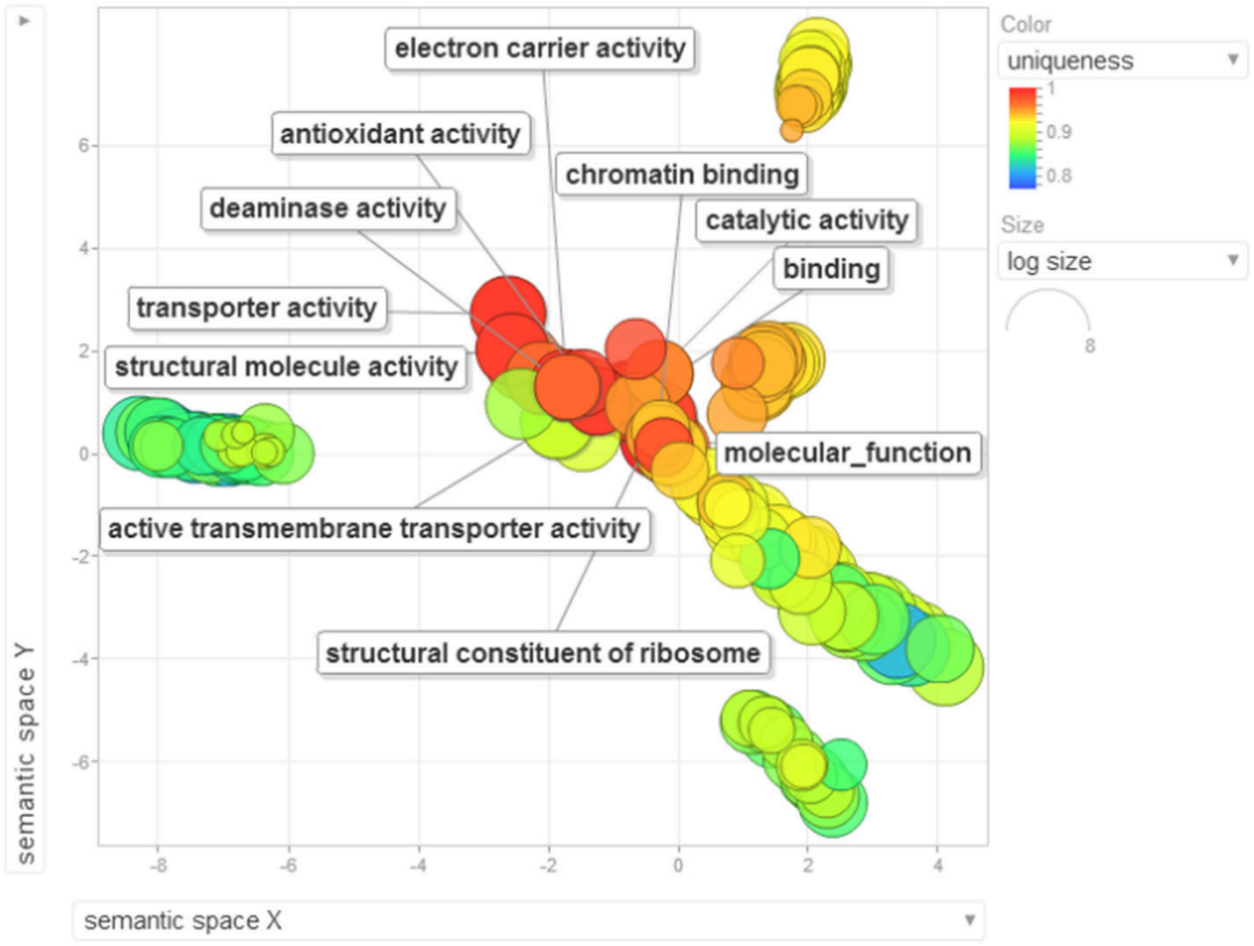
**The scatterplot visualizes the molecular function terms identified for the 532 proteins of black pepper**. The bubble color indicates the uniqueness of the GO terms (the legends for the first 20 terms have been included in the figure). A detailed list of GO terms along with their uniqueness has been supplied as a Table S5C.

Gene ontology terms were extensively reviewed to understand the proteome dataset. Initially, a second level GO analysis carried out for 532 proteins (Figure 5) identified at least 52 biological process terms which primarily included biological process (21%), response to stress (13%), biosynthetic process (6%), photosynthesis (6%), small molecule metabolic process (5%), carbohydrate metabolic process (5%), generation of precursor metabolites and energy (5%), protein folding (4%), cellular nitrogen compound metabolic process (4%), catabolic process (3%), cellular amino acid metabolic process (3%), and anatomical structure development (3%). Cellular component ontology revealed 28 different GO term which include plastid (16%), extracellular region (9%), thylakoid (9%), cellular component (9%), cell wall (7%), plasma membrane (7%), cytosol (7%), mitochondrion (6%), protein complex (6%), vacuole (6%), nucleolus (4%), ribosome (4%), nucleus (2%), and golgi apparatus(2%). With respect to molecular functions, we identified 33 GO terms predominated by ion binding (29%), molecular function (17%), oxidoreductase activity (14%), RNA binding (5%), peptidase activity (4%), unfolded protein binding (4%), lyase activity (3%), transmembrane transporter activity (3%), and ATPase activity (2%). Using Microsoft Excel pivot table analysis, 1203 GO terms were unique among all the identified GO terms. We used semantic similarity-based scatterplots offered by ReViGO for visualization and evaluating the potential function of the unique GO terms identified for all the proteins. ReViGO functions by negating the functional redundancy of long GO term list by avoiding uninformative general GO terms and are represented along with semantic similarities. The unique GO terms identified were functionally categorized into 792 unique biological processes terms (Table S5A), 181 cellular component terms (Table S5B), and 223 molecular function terms (Table S5C). The GO terms represented unique biological process terms such as response to endogenous stimulus, carbon utilization, multicellular organism reproduction, growth, and regulation of symbiosis, encompassing mutualism through parasitism (Figure 6). Cellular component terms that were uniquely represented include extracellular region, plasmodesma, membrane, outer membrane, extracellular vesicular exosome, macromolecular complex and protein-DNA complex (Figure 7). Unique molecular functions identified for 223 GO terms include chromatin binding, structural constituent of ribosome, structural molecule activity, antioxidant activity, active transmembrane transporter activity, substrate-specific transmembrane transporter activity and deaminase activity (Figure 8).

Protein sequences were also analyzed for their localization into organelles using TargetP1.1 (Emanuelsson et al., 2007) online tool. Target P1.1 is a comprehensive protein sequence search for identification of signal peptides for their localization to either secretory pathway, mitochondria or chloroplasts based on their N-terminal sequence motifs. We predicted a chloroplast transit peptide for 20% of the proteins suggesting a chloroplast localization, a mitochondrial targeting peptide for 10% of the proteins with localization in the mitochondrion and 22% of proteins had a signal peptide for localization in secretory pathways (Tables S4A–C). Out of 532, protein sequences identified, more than 48% (257 proteins) were predicted to localize in any other location (Table S4D).

Based on protein abundance ratio, *P. capsici* infection causes significant differential expression of host proteins which were involved in plant defense mechanisms. Some of the major proteins which were significantly down-regulated and were previously reported to have role in plant defense responses include subtilisin-like protease (Tian et al., 2007), catalases (Le Fevre et al., 2015), mannose glucose-specific lectin family protein (Xiang et al., 2011), rhicadhesin receptor-like protein (Ali et al., 2014), peptidyl-prolyl cis-trans isomerase 1-like protein (Kaur et al., 2010), germin-like protein (Manosalva et al., 2009), luminal binding protein (Galeano et al., 2015),oxygen-evolving enhancer protein, ribonuclease (Comella et al., 2008), aspartic proteinase nepenthesin-1 (Kadek et al., 2014), phosphoribulokinase, serine carboxypeptidase (Mugford et al., 2009), lipid transfer protein (Petti et al., 2010), glutathione s-transferase (Matern et al., 2015), polygalacturonase-inhibiting protein (Nguema-Ona et al., 2013),calnexin homolog (Howell, 2013), fasciclin-like arabinogalactan protein (Wang et al., 2014), xyloglucan-specific fungal endoglucanase inhibitor protein (Choi et al., 2013), glycine-rich protein (Kim and Buell, 2015), polyphenol oxidase (Llorente et al., 2014), and ubiquitin (He et al., 2015).

The complete list of proteins identified in the study with individual protein sequence name, BLAST2GO protein descriptions identified on *Viridiplantae* and *A. thaliana* TAIR10 databases, the PLGS score which corresponds to the confidence level for identification of protein, the protein abundance ratio (test vs. control) of differentially expressed protein, *P*-value identified for each hit and TargetP1.1 predicted localization have been described in the supporting tables (Table S2). The minimum expect value obtained by BLASTP analysis, mean percentage similarity of the protein, number of GO terms identified for each hit, individual GO terms for each hit, EC and InterProScan identification numbers along with sequence name and sequence description based on *Viridiplantae* database and *A. thaliana* database have been described in supporting tables (Tables S6, S7).

### Enrichment analysis of differentially down-regulated proteins

**Figure 9:**
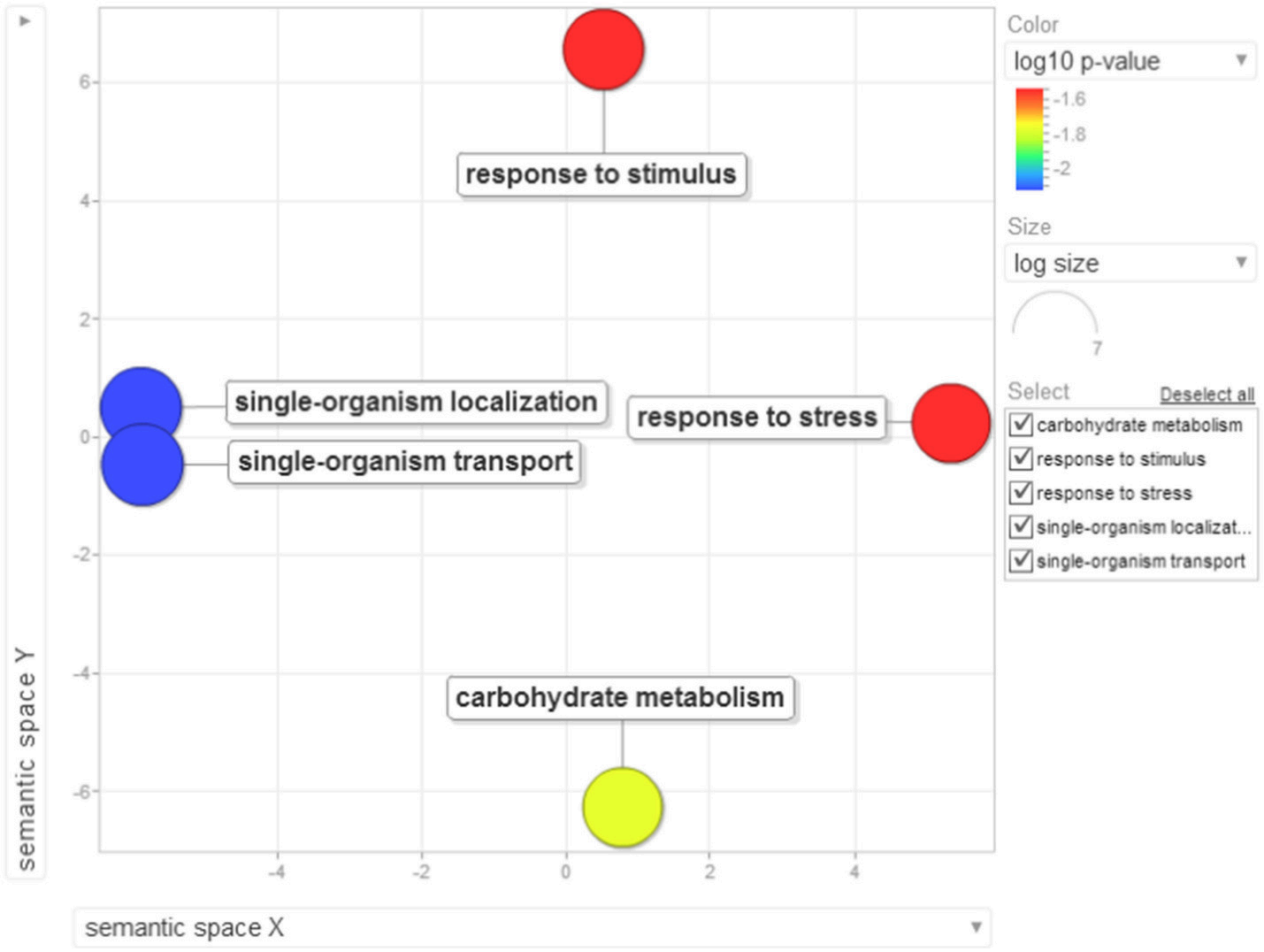
**Visualization of biological process terms of differentially down-regulated proteins identified based on enrichment analysis**. Bubble color denotes the *p*-value (legend provided in the image); bubble size indicates the frequency of the GO term in the underlying GOA database. List of GO terms representing the biological process along with *p*-values has been added as a Table S8A.

Earlier studies have shown that *Phytophthora*-host interactions demonstrate suppression of host immune responses where the pathogen targets the early innate immune components (Schlink, 2010). In our study, we found evidence that there was a dramatic down-regulation of more than 70% of the proteins. In order to evaluate and test this observation, we used the functional enrichment test which was carried out between low abundance proteins, their GO terms and their KEGG pathway over-representation using the Blast2GO application already available with the tool. The Fischer's extract test on the 134 differentially down-regulated proteins was found to be significant when compared with the reference protein dataset. The major KEGG pathways which suggested striking down-regulation in black pepper during *Phytophthora* infection include several primary and secondary metabolic pathways. Some of the major pathways include carbon fixation in photosynthetic organism, glycolysis/gluconeogenesis, citrate cycle, pentose phosphate pathway, cyano-amino acid metabolism, fructose and mannose metabolism, purine metabolism, glycine, serine and threonine metabolism, glutathione metabolism, starch and sucrose metabolism, phenylpropanoid biosynthesis, and other glycan degradation. The suppression of genes involved in photosynthesis, carbon fixation, and secondary metabolites involved in the biosynthesis of phytohormones during a compatible interaction have earlier been reported in *P. infestans*–potato pathosystem (Restrepo et al., 2005). The role of metabolic processes such as glycolysis/gluconeogenesis and sugar metabolism have earlier been reported to play an important role in the establishment of infection of oomycetes within a host. The stored nutrients serves as a major source of energy for *Phytophthora* during the initial stages of infection, but soon the pathogen starts utilizing host protein components (Hosseini et al., 2012). Out data suggest that *Phytophthora* dramatically affects source-sink dynamics which may lead to the collapse of the tissue leading to cell death and necrosis. Presently, the reasons for the suppression of these key pathways during the interaction of *P. capsici* and black pepper is unknown. Recent high-throughput studies on tomato–*P. capsici* pathosystem (Jupe et al., 2013) have suggested that there occur dramatic changes in primary and secondary metabolism in the tomato transcripts during the rapid lifecycle of this dreaded pathogen. *P. capsici*, being an oomycete, contains an arsenal of effector proteins which can take control of the host proteome for its own survival. Lack of knowledge of most of these effectors is another major challenge to study any of the affected phytopathosystems. The enrichment of gene ontology terms suggested an enrichment of 43 GO terms. These terms were visualized by two-dimensional semantic similarity-based scatterplots provided by ReViGO as explained earlier. Six major biological processes were significantly down-regulated during *Phytophthora* infection (Figure 9).

**Figure 10:**
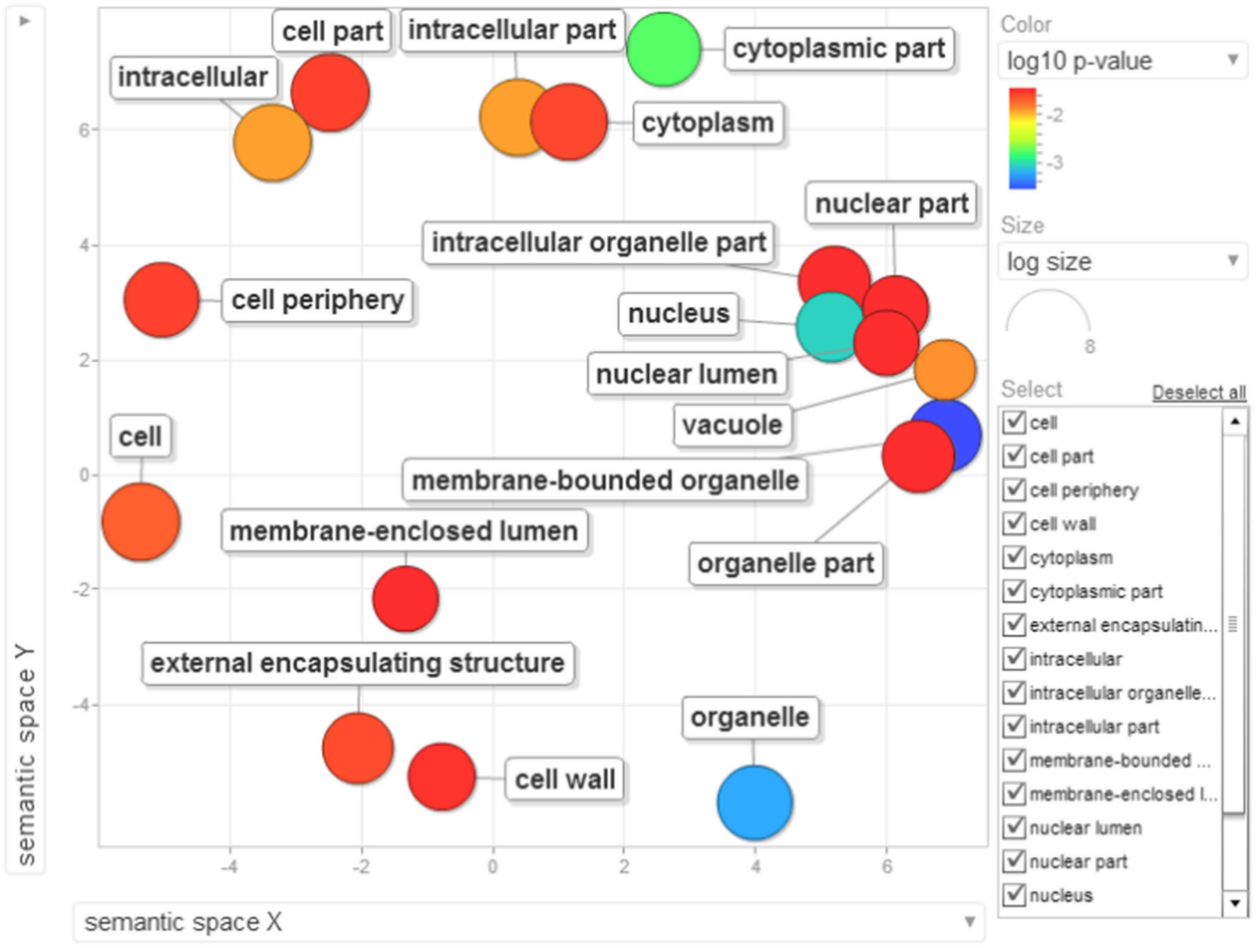
**Visualization of cellular component terms of differentially down-regulated proteins identified based on enrichment analysis**. Bubble color denotes the *p*-value (legend provided in the image); bubble size indicates the frequency of the GO term in the underlying GOA database. List of GO terms representing the cellular components along with *p*-values has been added as Table S8B.

**Figure 11:**
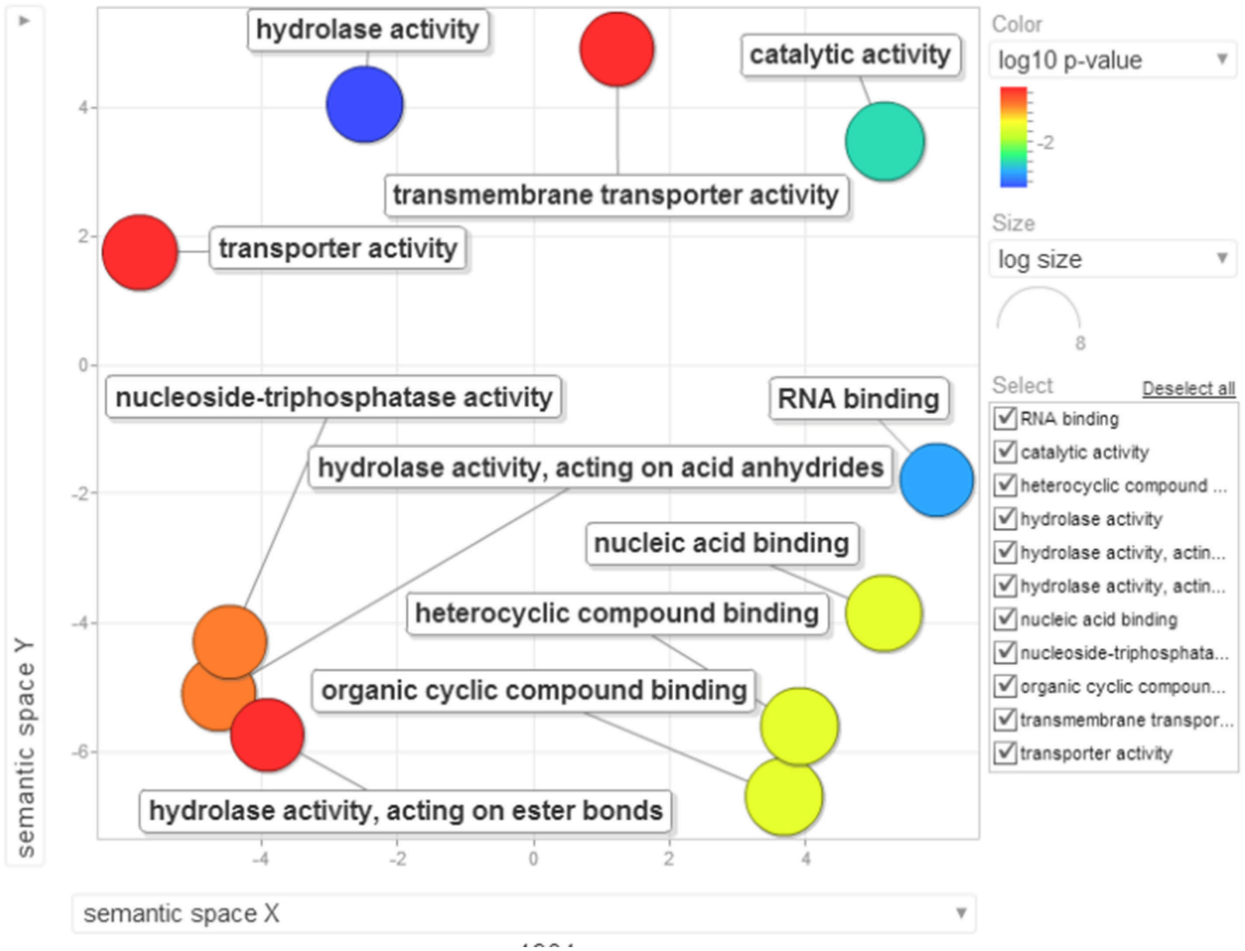
**Visualization of molecular function terms of differentially down-regulated proteins identified based on enrichment analysis**. Bubble color denotes the *p*-value (legend provided in the image); bubble size indicates the frequency of the GO term in the underlying GOA database. List of GO terms representing the molecular functions along with *p*-values has been added as a Table S8C.

Several previous have reported gene enrichment of terms such as carbohydrate metabolic process (Tauzin and Giardina, 2015), response to stress, transmembrane transport and response to stimulus (Li et al., 2016). These observations are also in consensus with recent high-through studies involving other oomycete phytopathosystems (Jupe et al., 2013; Zuluaga et al., 2015). At least 23 cellular components were involved during the overhaul of host proteins during *Phytophthora* infection (Figure 10) which include nucleus, intracellular membrane-bounded organelle, external encapsulating structure and cell wall. Moreover, 14 unique molecular functions were strikingly involved during the susceptibility of black pepper to *P. capsici* (Figure 11).

Role for RNA binding and nucleic acid binding protein (Qi et al., 2010), hydrolase activity (Kong et al., 2015), and ATPase activity (Chen et al., 2010) are active areas of research in understanding the plant immunity. These observations need to be validated by further research.

In addition to the BLAST2GO enrichment analysis, we re-evaluated the data using another tool hosted as KOBAS 2.0. The enrichment revealed significant pathways such as carbon fixation in photosynthetic organisms, photosynthesis, carbon metabolism, protein processing in the endoplasmic reticulum, glyoxylate and dicarboxylate metabolism, s-adenosyl-L-methionine biosynthesis, glucose and xylose degradation, and ethylene biosynthesis (Table S8) involved during the susceptibility of black pepper to *Phytophthora*.

### Network analysis of significantly down-regulated proteins

**Figure 12:**
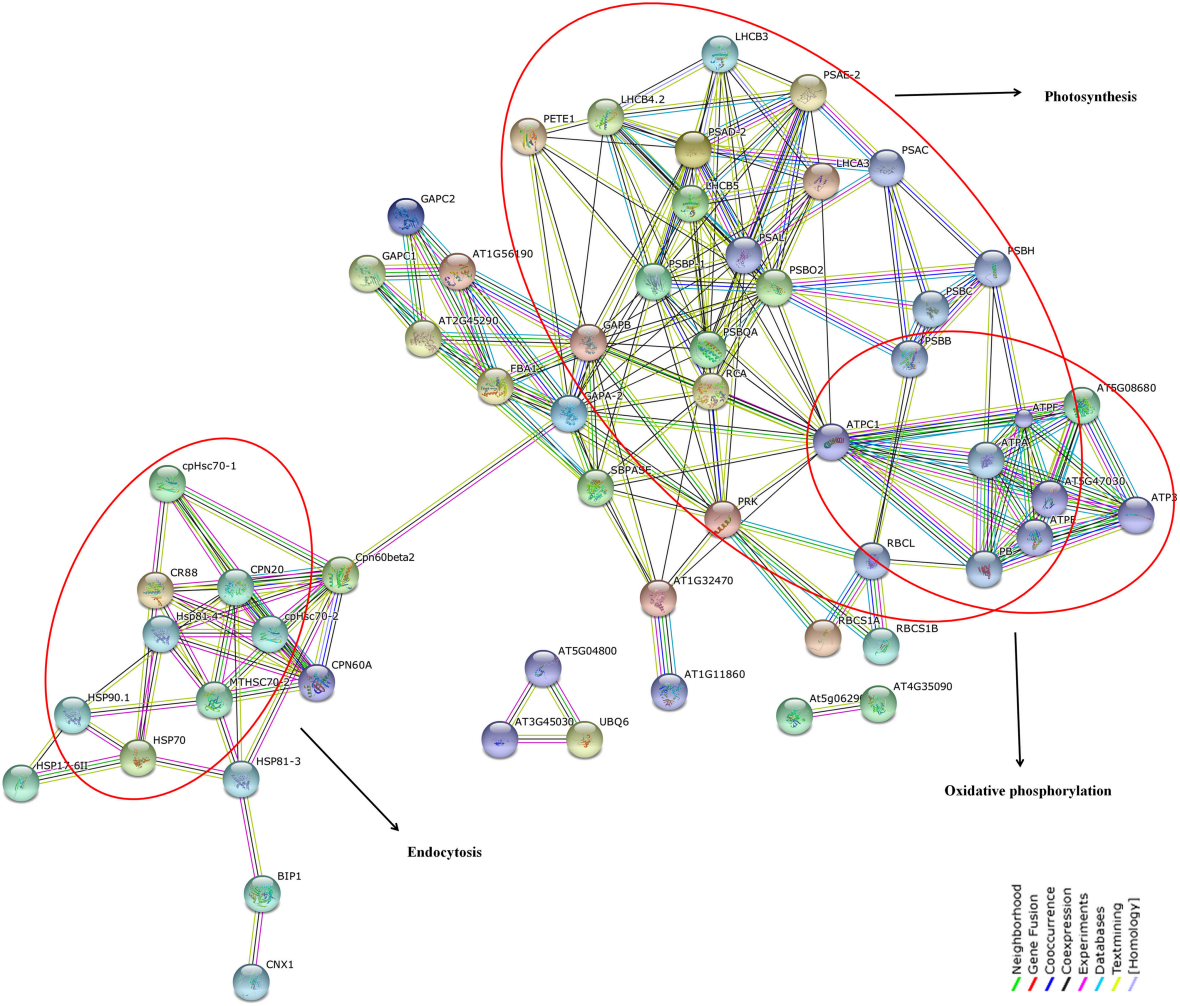
**Network interactions of differentially down-regulated proteins under different biological functions by STRING 9.1 analysis at a maximum confidence level of 0.9**. Over-representation of key pathways such as photosynthesis, oxidative phosphorylation, and endocytosis have been highlighted in red. List of protein nodes identified against *Arabidopsis thaliana* TAIR10 reference database have been given in Table S9.

Reconstruction and representation of plant cellular pathway based on expression profiling have been always challenging especially for non-model plants. We suppose that the susceptibility of black pepper to *Phytophthora* involves a complex interaction of proteins which are regulated and controlled by the host and pathogen. In order to test this, we have utilized a homology based prediction for identifying the protein-protein interactions among the differentially down-regulated proteins using STRING 9.1 online tool against *A. thaliana* TAIR10 database. This approach have earlier been highlighted in several high-throughput studies (Van Baarlen et al., 2008). At the highest confidence level of 0.9, we observed overrepresentation of protein interactions involved in photosynthesis, metabolic pathways, carbon fixation in photosynthetic organism, protein processing in endoplasmic reticulum, carbon metabolism, glycoxylate, and dicarboxylate metabolism, oxidative phosphorylation, endocytosis, and spliceosome (Figure 12). All the protein nodes generated using the protein dataset against *A. thaliana* database along with their corresponding STRING IDs and the abbreviations of these interacting proteins along with their predicted functional partners are provided in the supporting table (Table S9).

### Validation of label-free quantitative proteomics results

**Figure 13:**
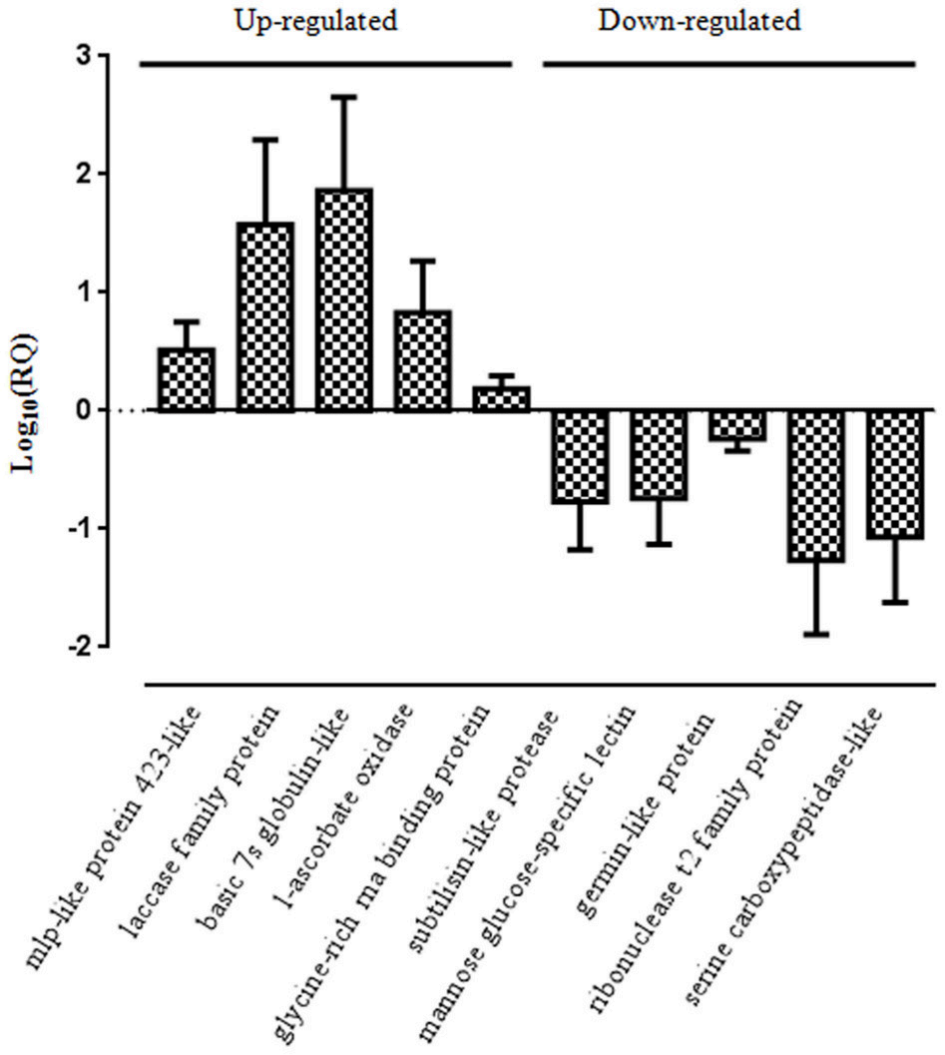
**Real-time PCR analysis of selected up- and down-regulated genes**. The qRT-PCR was conducted using gene-specific primers to analyze their relative expression in leaf samples. *Pn18srRNA* was used as an internal control to normalize the samples. Values are means of three biological replicates performed in triplicate.

We validated the label-free quantitative proteomics results obtained using a quantitative real-time RT-PCR analysis. Five genes were randomly selected from up-regulated protein dataset which include *mlp-like protein* (comp59353_c0_seq1_1), *laccase family protein* (comp77477_c0_seq1_2), *basic 7s globulin-like protein* (comp87186_c0_seq1_2), *l-ascorbate oxidase homolog* (comp51029_c0_seq1_2), and *glycine-rich RNA binding protein* (comp51427_c0_seq2_2). We also selected five genes from the list of down-regulated protein dataset which include *subtilisin-like protease* (comp87657_c0_seq1_4), *mannose glucose-specific lectin family protein* (comp67436_c0_seq1_1), *germin-like protein* (comp24430_c0_seq1_2), *ribonuclease t2 family protein* (comp73900_c0_seq1_3), and *serine carboxypeptidase protein* (comp88598_c0_seq1_3). Transcript abundance was compared between mock control and *P. capsici* infected (24 hpi) samples in biological replicates. The quantitative expression of selected genes was represented as log_10_ relative quantity (RQ) of target mRNA normalized against *Pn18srRNA* reference gene (Figure 13). The qRT-PCR results of selected up- or down- regulated genes appreciably correlated with the label-free quantitative proteomics results. Up-regulation of several novel genes such as *mlp-like proteins* which are pathogenesis-related proteins (Bufe et al., 1996), glutathione s-transferases (Dean et al., 2005), polyphenol oxidases which are regulated during development of resistance against pearl millet downy mildew disease (Raj et al., 2006), laccase which regulate lignification during fungal infection (Mayer and Staples, 2002), globulin-like proteins and ascorbate oxidases reported to be involved in black spot discoloration in wheat (Mak et al., 2006) clearly suggest that the interaction of *P. capsici* with *P. nigrum* does involve a dramatic regulation of host proteome as well as transcriptome. It is surprising to observe that even with the up-regulation of several anti-fungal proteins like aspartyl protease (Xia et al., 2004), pectinesterase like protein (An et al., 2008), polyphenol oxidase and mlp-like proteins does not prevent the susceptibility of the host to the oomycete pathogen. This may be attributed to down-regulation of many of the major proteins involved in primary and secondary metabolism (Table S2A). Major down-regulation of key plant immune responsive components like subtilisin-like protease (Figueiredo et al., 2014), serine carboxypeptidase which are required in anti-microbial compound synthesis leading to defense (Mugford and Milkowski, 2012), mannose-glucose specific lectins (Xiang et al., 2011), and germin-like proteins which gives broad-spectrum disease resistance (Manosalva et al., 2009) clearly suggest that this phytopathosystem is unique and highly complex warranting further investigation.

### Full-length gene identification, cloning, and sequence characterization of novel genes

**Figure 14:**
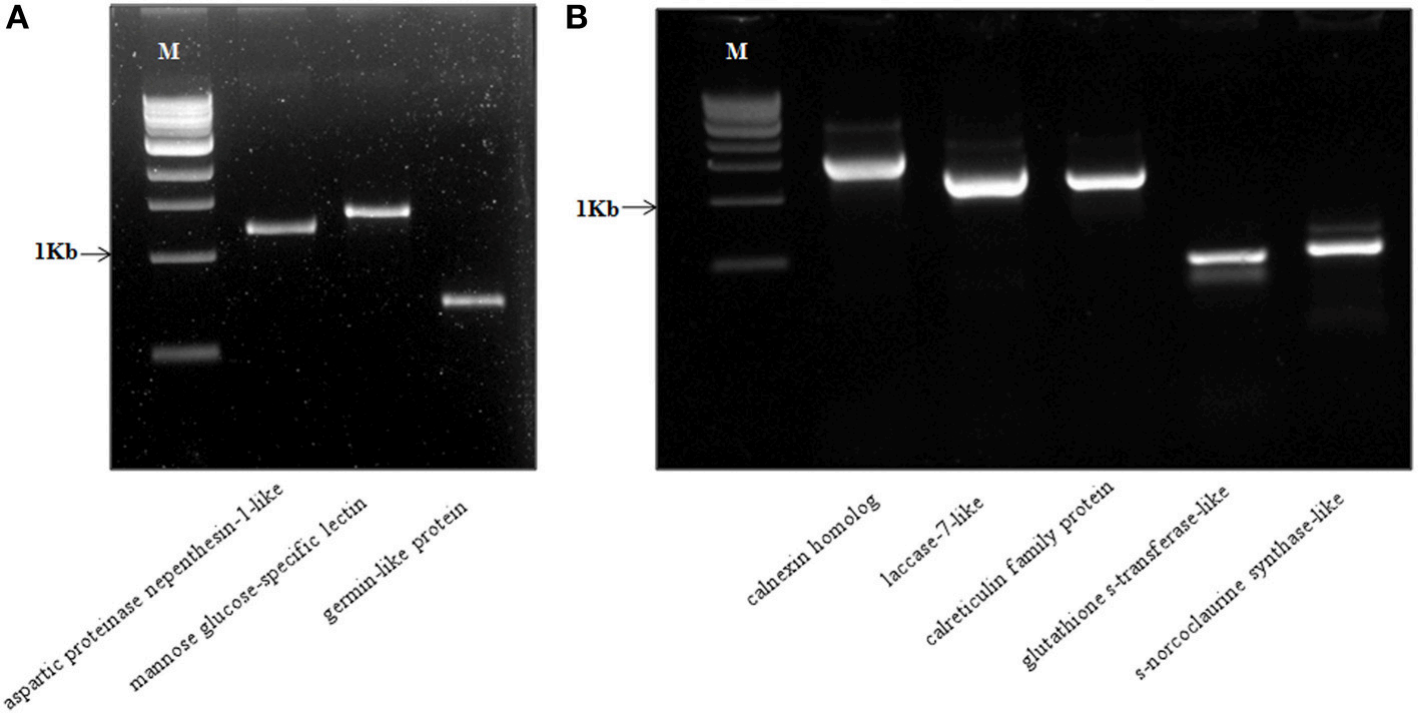
**(A,B)** Detection of a full-length amplicon of selected genes amplified from cDNA synthesized from *Piper nigrum* by RT-PCR. The amplicon size predicted along with putative names have been represented on each line. M- 1 Kbp ladder (NEB). The primers used along with all necessary detail have been listed in the Table S10.

In our study, we also report a novel strategy for identification of full-length genes of the corresponding protein candidates identified using a “reverse-proteomics” approach (Walhout and Vidal, 2001). Using the protein sequence name as identifiers, we manually derived the correct nucleotide coding sequences of at least 526 protein hits which were within the *P. nigrum* transcriptome dataset. We further validated this strategy by designing full-length cloning primers for eight randomly selected genes with a range of different molecular weights (included in parenthesis). The genes selected include *aspartic proteinase nepenthesin-1 protein* (comp66934_c0_seq1_2; MW–1211 bp), *mannose glucose-specific lectin family protein* (comp67436_c0_seq1_1; MW–1356 bp), *germin-like protein* (comp24430_c0_seq1_2; MW–711 bp), *calnexin homolog protein* (comp85398_c0_seq1; MW–1632 bp), *laccase-7-like protein* (comp77477_c0_seq1; MW–1305 bp), *calreticulin family protein*(comp77065_c0_seq2; MW–1254 bp), *glutathione s-transferase-like protein* (comp72509_c0_seq2; MW–456 bp), and *s-norcoclaurine synthase-like protein* (comp62928_c0_seq1; MW–540 bp). We were able to successfully amplify all the predicted genes with expected molecular weight (Figure 14). These genes were further cloned into pGEM T-easy TA vector (Promega Inc.), sequenced using Sanger sequencing available at Rajiv Gandhi Center for Biotechnology, Thiruvananthapuram, India and analyzed using NCBI BLASTN analysis for confirmation of the gene predicted. This strategy is highly efficient for studying the molecular components of non-model plants and would certainly pave the way for further research in diverse plant groups including black pepper. All the primers used in the current study have been listed with necessary information in the supporting table (Table S10).

## Conclusion

In the present study, we report a comprehensive high-throughput proteomics tool for understanding the plant-oomycete interaction. We explore the use of a transcriptome assisted label-free quantitative proteomics approach for identification of novel leaf proteome of black pepper when challenged with *P. capsici*. Black pepper has been susceptible to *Phytophthora* foot rot for over a century. There are no varieties of black pepper which can completely resist this disease and the progress in understanding the molecular components of this phytopathosystem has been severely hampered due to lack of proteome or transcriptome databases (Gordo et al., 2012). In our study, we describe for the first time, a complete list of 532 proteins of black pepper derived from a public transcriptome dataset generated using black pepper leaves (Table S11). We have utilized BLAST2GO tool for thorough annotation of over 518 identified proteins. We further report a total of 526 novel open reading frames of black pepper effectively identified through a reverse proteomic approach (Table S12) (Walhout and Vidal, 2001). These genes can be directly utilized for studies including cloning, expression, and further downstream characterization. This study provides comprehensive insights into the complex network of proteome changes that happen in black pepper during a hemibiotrophic oomycete infection.

## Author contributions

CM, MS—conceived and designed the experiments; CM, AK, GS—performed the experiments; CM, AS—analyzed the data; AJ, AS—contributed reagents/materials/analysis tools: CM, MS—wrote the paper.

### Conflict of interest statement

The authors declare that the research was conducted in the absence of any commercial or financial relationships that could be construed as a potential conflict of interest.

## References

[B1] Ali A. Alexandersson E. Sandin M. Resjö S. Lenman M. Hedley P.. (2014). Quantitative proteomics and transcriptomics of potato in response to Phytophthora infestans in compatible and incompatible interactions. BMC Genomics. 15:497. 10.1186/1471-2164-15-497 24947944 PMC4079953

[B2] An S. Choi H. Lee (2008). Pepper pectin methylesterase inhibitor protein CaPMEI1 is required for antifungal activity, basal disease resistance and abiotic stress tolerance. Planta 228, 61–78. 10.1007/s00425-008-0719-z 18327607 PMC2413075

[B3] Anandaraj Sarma (1995). Diseases of black pepper (*Piper nigrum* L.) and their management. J. Spices Aromat. Crops 4, 17–23.

[B4] Asha S. Nisha J. Soniya E. (2012). *In silico* characterisation and phylogenetic analysis of two evolutionarily conserved miRNAs (miR166 and miR171) from black pepper (*Piper nigrum* L.). Plant Mol. Biol. Rep. 31, 707–718. 10.1007/s11105-012-0532-5

[B5] Ashburner M. Ball C. A. Blake J. A. Botstein D. Butler H. Cherry J. M.. (2000). Gene ontology: tool for the unification of biology. Nat. Genet. 25, 25–29. 10.1038/75556 10802651 PMC3037419

[B6] Balmer A. Pastor V. Gamir J. Flors V. Mauch-Mani B. (2015). The “prime-ome”: towards a holistic approach to priming. Trends Plant Sci. 20, 443–452. 10.1016/j.tplants.2015.04.002 25921921

[B7] Bradford M. M. (1976). A rapid and sensitive method for the quantitation of microgram quantities of protein utilizing the principle of protein-dye binding. Anal. Biochem. 72, 248–254. 10.1016/0003-2697(76)90527-3 942051

[B8] Bufe S. Kahlert Schlaak (1996). The major birch pollen allergen, Bet v 1, shows ribonuclease activity. Planta 199, 413–415. 10.1007/BF00195733 8771801

[B9] Chen X. Chern M. Canlas P. E. Ruan D. Jiang C. Ronald P. C. (2010). An ATPase promotes autophosphorylation of the pattern recognition receptor XA21 and inhibits XA21-mediated immunity. Proc. Natl. Acad. Sci. U.S.A. 107, 8029–8034. 10.1073/pnas.0912311107 20385831 PMC2867851

[B10] Choi H. W. Kim N. H. Lee Y. K. Hwang B. K. (2013). The pepper extracellular xyloglucan-specific endo-β-1, 4-glucanase inhibitor protein gene, CaXEGIP1, is required for plant cell death and defense responses. Plant Physiol. 161, 384–396. 10.1104/pp.112.203828 23093361 PMC3532269

[B11] Chung C.-L. L. Longfellow J. M. Walsh E. K. Kerdieh Z. Esbroeck G. Van Balint-Kurti P.. (2010). Resistance loci affecting distinct stages of fungal pathogenesis: use of introgression lines for QTL mapping and characterization in the maize–Setosphaeria turcica pathosystem. BMC Plant Biol. 10:103. 10.1186/1471-2229-10-103 20529319 PMC3017769

[B12] Comella P. Pontvianne F. Lahmy S. Vignols F. Barbezier N. Debures A.. (2008). Characterization of a ribonuclease III-like protein required for cleavage of the pre-rRNA in the 3′ ETS in Arabidopsis. Nucleic Acids Res. 36, 1163–1175. 10.1093/nar/gkm1130 18158302 PMC2275086

[B13] Conesa A. Götz S. García-Gómez J. Terol J. Talón M. Robles M. (2005). Blast2GO: a universal tool for annotation, visualization and analysis in functional genomics research. Bioinformatics 21, 3674–3676. 10.1093/bioinformatics/bti610 16081474

[B14] Dean J. D. Goodwin P. H. Hsiang T. (2005). Induction of glutathione S-transferase genes of Nicotiana benthamiana following infection by *Colletotrichum destructivum* and *C. orbiculare* and involvement of one in resistance. J. Exp. Bot. 56, 1525–1533. 10.1093/jxb/eri145 15837710

[B15] Emanuelsson B. von Heijne Nielsen (2007). Locating proteins in the cell using TargetP, SignalP and related tools. Nat. Protoc. 2, 953–971. 10.1038/nprot.2007.131 17446895

[B16] Evans V. Barker G. Heesom K. Fan J. Bessant C. Matthews D. (2012). *De novo* derivation of proteomes from transcriptomes for transcript and protein identification. Nat. Methods 9, 1207–1211. 10.1038/nmeth.2227 23142869 PMC3581816

[B17] Figueiredo A. Monteiro F. Sebastiana M. (2014). Subtilisin-like proteases in plant–pathogen recognition and immune priming: a perspective. Front. Plant Sci. 5:739. 10.3389/fpls.2014.00739 25566306 PMC4271589

[B18] Fisher M. C. Henk D. A. Briggs C. J. Brownstein J. S. Madoff L. C. McCraw S. L.. (2012). Emerging fungal threats to animal, plant and ecosystem health. Nature 484,186–194. 10.1038/nature10947 22498624 PMC3821985

[B19] Franceschini A. Szklarczyk D. Frankild S. Kuhn M. Simonovic M. Roth A.. (2013). STRING v9.1: protein-protein interaction networks, with increased coverage and integration. Nucleic Acids Res. 41, D808–D815. 10.1093/nar/gks1094 23203871 PMC3531103

[B20] Galeano E. Vasconcelos T. S. Vidal M. Mejia-Guerra M. K. Carrer H. (2015). Large-scale transcriptional profiling of lignified tissues in Tectona grandis. BMC Plant Biol. 15:221. 10.1186/s12870-015-0599-x 26369560 PMC4570228

[B21] Gopinath V. Raghunandanan S. Gomez R. Jose L. Surendran A. Ramachandran R.. (2015). Profiling the proteome of Mycobacterium tuberculosis during dormancy and reactivation. Mol. Cell Proteomics 14, 2160–2176. 10.1074/mcp.M115.051151 26025969 PMC4528245

[B22] Gordo S. Pinheiro D. Moreira E. Rodrigues S. Poltronieri M. Lemos O.. (2012). High-throughput sequencing of black pepper root transcriptome. BMC Plant Biol. 12:168. 10.1186/1471-2229-12-168 22984782 PMC3487918

[B23] Götz García-Gómez, Terol,. (2008). High-throughput functional annotation and data mining with the Blast2GO suite. Nucleic Acids Res. 36, 3420–3435. 10.1093/nar/gkn176 18445632 PMC2425479

[B24] Grabherr M. Haas B. Yassour M. Levin J. Thompson D. Amit I.. (2011). Full-length transcriptome assembly from RNA-Seq data without a reference genome. Nat. Biotechnol. 29, 644–652. 10.1038/nbt.1883 21572440 PMC3571712

[B25] He B. Tao X. Gu Y. Wei C. Cheng X. Xiao S.. (2015). Transcriptomic analysis and the expression of disease-resistant Genes in Oryza meyeriana under native condition. PLoS ONE 10:e0144518. 10.1371/journal.pone.0144518 26640944 PMC4671656

[B26] Hosseini S. Karlsson M. Jensen D. F. Heyman F. (2012). Quantification of Phytophthora pisi DNA and RNA transcripts during in planta infection of pea. Eur. J. Plant Pathol. 132, 455–468. 10.1007/s10658-011-9890-3

[B27] Howell S. H. (2013). Endoplasmic reticulum stress responses in plants. Annu. Rev. Plant Biol. 64, 477–499. 10.1146/annurev-arplant-050312-120053 23330794

[B28] Isaacson T. Damasceno C. M. Saravanan R. S. He Y. Catalá C. Saladié M.. (2006). Sample extraction techniques for enhanced proteomic analysis of plant tissues. Nat. Protoc. 1, 769–774. 10.1038/nprot.2006.102 17406306

[B29] Jones J. D. Dangl J. L. (2006). The plant immune system. Nature 444, 323–329. 10.1038/nature05286 17108957

[B30] Joy N. Asha S. Mallika V. Soniya E. (2013). *De novo* transcriptome sequencing reveals a considerable bias in the incidence of simple sequence repeats towards the downstream of “Pre-miRNAs” of Black Pepper. PLoS ONE 8:e56694. 10.1371/journal.pone.0056694 23469176 PMC3587635

[B31] Jupe J. Stam R. Howden A. Morris J. Zhang R. Hedley P.. (2013). *Phytophthora capsici*-tomato interaction features dramatic shifts in gene expression associated with a hemi-biotrophic lifestyle. Genome Biol. 14:R63. 10.1186/gb-2013-14-6-r63 23799990 PMC4054836

[B32] Kadek A. Tretyachenko V. Mrazek H. Ivanova L. Halada P. Rey M.. (2014). Expression and characterization of plant aspartic protease nepenthesin-1 from *Nepenthes gracilis*. Protein Expr. Purif. 95, 121–128. 10.1016/j.pep.2013.12.005 24365662

[B33] Kaur P. Jost R. Sivasithamparam K. Barbetti M. J. (2010). Proteome analysis of the *Albugo candida*–*Brassica juncea* pathosystem reveals that the timing of the expression of defence-related genes is a crucial determinant of pathogenesis. J. Exp. Bot. 62, 1285–1298. 10.1093/jxb/erq365 21193577 PMC3022411

[B34] Kim J. Buell C. R. (2015). A revolution in plant metabolism: Genome-enabled pathway discovery. Plant Physiol. 169, 1532–1539. 10.1104/pp.15.00976 26224805 PMC4634072

[B35] Kong G. Zhao Y. Jing M. Huang J. Yang J. Xia Y.. (2015). The activation of phytophthora effector Avr3b by plant cyclophilin is required for the nudix hydrolase activity of Avr3b. PLoS Pathog. 11:e1005139. 10.1371/journal.ppat.1005139 26317500 PMC4552650

[B36] Kottapalli K. Zabet-Moghaddam M. Rowland D. Faircloth W. Mirzaei M. Haynes P.. (2013). Shotgun label-free quantitative proteomics of water-deficit-stressed midmature peanut (*Arachis hypogaea* L.) seed. J. Proteome Res. 12, 5048–5057. 10.1021/pr400936d 24094305

[B37] Krishnan A. Mahadevan C. Mani T. Sakuntala M. (2015). Virus-induced gene silencing (VIGS) for elucidation of pathogen defense role of serine/threonine protein kinase in the non-model plant Piper colubrinum Link. Plant Cell Tissue Organ Cult. 122, 269–283. 10.1007/s11240-015-0764-9

[B38] Lamour K. Stam R. Jupe J. Huitema E. (2012). The oomycete broad-host-range pathogen *Phytophthora capsici*. Mol. Plant Pathol. 13, 329–337. 10.1111/j.1364-3703.2011.00754.x 22013895 PMC6638677

[B39] Le Berre J. Y. Engler G. Panabières F. (2008). Exploration of the late stages of the tomato–Phytophthora parasitica interactions through histological analysis and generation of expressed sequence tags. New Phytol. 177, 480–492. 10.1111/j.1469-8137.2007.02269.x 18028297

[B40] Le Fevre R. Evangelisti E. Rey T. Schornack S. (2015). Modulation of host cell biology by plant pathogenic microbes. Annu. Rev. Cell Dev. Biol. 31, 201–229. 10.1146/annurev-cellbio-102314-112502 26436707

[B41] Li B. Meng X. Shan L. He P. (2016). Transcriptional regulation of pattern-triggered immunity in plants. Cell Host Microbe. 19, 641–650. 10.1016/j.chom.2016.04.011 27173932 PMC5049704

[B42] Llorente B. López M. G. Carrari F. Asís R. Naranjo R. D. D. P. Flawiá M. M. (2014). Downregulation of polyphenol oxidase in potato tubers redirects phenylpropanoid metabolism enhancing chlorogenate content and late blight resistance. Mol. Breed. 34, 2049–2063. 10.1007/s11032-014-0162-8

[B43] Luge T. Kube M. Freiwald A. Meierhofer D. Seemüller E. Sauer S. (2014). Transcriptomics assisted proteomic analysis of *Nicotiana occidentalis* infected by Candidatus Phytoplasma mali strain AT. Proteomics 14, 1882–1889. 10.1002/pmic.201300551 24920314

[B44] Luzzatto-Knaan T. Kerem Z. Doron-Faigenboim A. Yedidia I. (2014). Priming of protein expression in the defence response of Zantedeschia aethiopica to Pectobacterium carotovorum. Mol. Plant Pathol. 15, 364–378. 10.1111/mpp.12100 24822269 PMC6638884

[B45] Mahadevan C. Jaleel A. Deb L. Thomas G. Sakuntala M. (2014). Development of an efficient virus induced gene silencing strategy in the non-model wild ginger-*Zingiber zerumbet* and investigation of associated proteome changes. PLoS ONE 10:e0124518. 10.1371/journal.pone.0124518 25918840 PMC4412686

[B46] Mak Y. Willows R. D. Roberts T. H. Wrigley C. W. Sharp P. J. Copeland L. (2006). Black Point is associated with reduced levels of stress, disease-and defence-related proteins in wheat grain. Mol. Plant Pathol. 7, 177–189. 10.1111/j.1364-3703.2006.00330.x 20507438

[B47] Manosalva D. Liu Zhu (2009). A germin-like protein gene family functions as a complex quantitative trait locus conferring broad-spectrum disease resistance in rice. Plant Physiol. 149, 286–296. 10.1104/pp.108.128348 19011003 PMC2613727

[B48] Matern S. Peskan-Berghoefer T. Gromes R. Kiesel R. V. Rausch T. (2015). Imposed glutathione-mediated redox switch modulates the tobacco wound-induced protein kinase and salicylic acid-induced protein kinase activation state and impacts on defence against *Pseudomonas syringae*. J. Exp. Bot. 66, 1935–1950. 10.1093/jxb/eru546 25628332 PMC4378631

[B49] Mayer Staples,. (2002). Laccase: new functions for an old enzyme. Phytochemistry 60, 551–565. 10.1016/S0031-9422(02)00171-1 12126701

[B50] Meng Y. Liu F. Pang C. Fan S. Song M. Wang D.. (2011). Label-free quantitative proteomics analysis of cotton leaf response to nitric oxide. J. Proteome Res. 10, 5416–5432. 10.1021/pr200671d 22029526

[B51] Mochida K. Shinozaki K. (2011). Advances in omics and bioinformatics tools for systems analyses of plant functions. Plant Cell Physiol. 52, 2017–2038. 10.1093/pcp/pcr153 22156726 PMC3233218

[B52] Moy P. Qutob D. Chapman B. P. Atkinson I. Gijzen M. (2004). Patterns of gene expression upon infection of soybean plants by *Phytophthora sojae*. Mol. Plant Microbe Interact. 17, 1051–1062. 10.1094/MPMI.2004.17.10.1051 15497398

[B53] Mugford S. Milkowski C. (2012). Serine carboxypeptidase-like acyltransferases from plants. Meth. Enzymol. 516, 279–297. 10.1016/B978-0-12-394291-3.00006-X 23034234

[B54] Mugford S. T. Qi X. Bakht S. Hill L. Wegel E. Hughes R. K.. (2009). A serine carboxypeptidase-like acyltransferase is required for synthesis of antimicrobial compounds and disease resistance in oats. Plant Cell 21, 2473–2484. 10.1105/tpc.109.065870 19684243 PMC2751944

[B55] Nguema-Ona E. Moore J. P. Fagerström A. D. Fangel J. U. Willats W. G. Hugo A.. (2013). Overexpression of the grapevine PGIP1 in tobacco results in compositional changes in the leaf arabinoxyloglucan network in the absence of fungal infection. BMC Plant Biol. 13:46. 10.1186/1471-2229-13-46 23506352 PMC3621556

[B56] Niehl A. Zhang Z. Kuiper M. Peck S. Heinlein M. (2013). Label-free quantitative proteomic analysis of systemic responses to local wounding and virus infection in *Arabidopsis thaliana*. J. Proteome Res. 12, 2491–2503. 10.1021/pr3010698 23594257

[B57] Petti C. Khan M. Doohan F. (2010). Lipid transfer proteins and protease inhibitors as key factors in the priming of barley responses to Fusarium head blight disease by a biocontrol strain of Pseudomonas fluorescens. Funct. Integr. Genomics 10, 619–627. 10.1007/s10142-010-0177-0 20526726

[B58] Qi Y. Tsuda K. Joe A. Sato M. Nguyen L. V. Glazebrook J.. (2010). A putative RNA-binding protein positively regulates salicylic acid-mediated immunity in Arabidopsis. Mol. Plant Microbe Interact. 23, 1573–1583. 10.1094/MPMI-05-10-0106 20636102

[B59] Raj S. N. Sarosh B. R. Shetty (2006). Induction and accumulation of polyphenol oxidase activities as implicated in development of resistance against pearl millet downy mildew disease. Funct. Plant Biol. 33, 563–571. 10.1071/FP06003 32689264

[B60] Restrepo S. Myers K. L. Del Pozo O. Martin G. B. Hart A. L. Buell C. R.. (2005). Gene profiling of a compatible interaction between Phytophthora infestans and *Solanum tuberosum* suggests a role for carbonic anhydrase. Mol. Plant Microbe Interact. 18, 913–922. 10.1094/MPMI-18-0913 16167762

[B61] Rice P. Longden I. Bleasby A. (2000). EMBOSS: the European molecular biology open software suite. Trends Genet. 16, 276–277. 10.1016/S0168-9525(00)02024-2 10827456

[B62] Schlink K. (2010). Down-regulation of defense genes and resource allocation into infected roots as factors for compatibility between *Fagus sylvatica* and *Phytophthora citricola*. Funct. Integr. Genomics. 10, 253–264. 10.1007/s10142-009-0143-x 19813036

[B63] Schmittgen T. Livak K. (2008). Analyzing real-time PCR data by the comparative CT method. Nat. Protoc. 3, 1101–1108. 10.1038/nprot.2008.73 18546601

[B64] Tauzin A. S. Giardina T. (2015). Sucrose and invertases, a part of the plant defense response to the biotic stresses. Front Plant Sci. 5:293. 10.3389/fpls.2014.00293 25002866 PMC4066202

[B65] Tian M. Win J. Song J. van der Hoorn R. van der Knaap E. Kamoun S. (2007). A Phytophthora infestans cystatin-like protein targets a novel tomato papain-like apoplastic protease. Plant Physiol. 143, 364–377. 10.1104/pp.106.090050 17085509 PMC1761951

[B66] Untergasser A. Nijveen H. Rao X. Bisseling T. Geurts R. Leunissen J. A. (2007). Primer3Plus, an enhanced web interface to Primer3. Nucleic Acids Res. 35, W71–W74. 10.1093/nar/gkm306 17485472 PMC1933133

[B67] Van Baarlen P. Van Esse H. P. Siezen R. J. Thomma B. P. (2008). Challenges in plant cellular pathway reconstruction based on gene expression profiling. Trends Plant Sci. 13, 44–50. 10.1016/j.tplants.2007.11.003 18155635

[B68] Vizcaíno J. A. Csordas A. del-Toro N. Dianes J. A. Griss J. Lavidas I.. (2016). 2016 update of the PRIDE database and its related tools. Nucleic Acids Res. 44, D447–D456. 10.1093/nar/gkv1145 26527722 PMC4702828

[B69] Walhout A. Vidal M. (2001). Protein interaction maps for model organisms. Nat. Rev. Mol. Cell Biol. 2, 55–63. 10.1038/35048107 11413466

[B70] Wang H. Jiang C. Wang C. Yang Y. Yang L. Gao X.. (2014). Antisense expression of the fasciclin-like arabinogalactan protein FLA6 gene in Populus inhibits expression of its homologous genes and alters stem biomechanics and cell-wall composition in transgenic trees. J. Exp. Bot. 66, 1291–1302. 10.1093/jxb/eru479 25428999 PMC4339592

[B71] Wang Y. A. N. Bouwmeester K. Mortel J. E. Shan W. Govers F. (2013). A novel Arabidopsis–oomycete pathosystem: differential interactions with *Phytophthora capsici* reveal a role for camalexin, indole glucosinolates and salicylic acid in defence. Plant Cell Environ. 36, 1192–1203. 10.1111/pce.12052 23237451

[B72] Weinhold A. Wielsch N. Svatoš A. Baldwin I. (2015). Label-free nanoUPLC-MSE based quantification of antimicrobial peptides from the leaf apoplast of *Nicotiana attenuata*. BMC Plant Biol. 15:18. 10.1186/s12870-014-0398-9 25604123 PMC4318441

[B73] Wiśniewski J. Zielinska D. Mann M. (2011). Comparison of ultrafiltration units for proteomic and N-glycoproteomic analysis by the filter-aided sample preparation method. Anal. Biochem. 410, 307–309. 10.1016/j.ab.2010.12.004 21144814

[B74] Xia S. Borevitz B. Guo (2004). An extracellular aspartic protease functions in Arabidopsis disease resistance signaling. EMBO J. 23, 980–988. 10.1038/sj.emboj.7600086 14765119 PMC380998

[B75] Xiang Y. Song M. Wei Z. Tong J. Zhang L. Xiao L.. (2011). A jacalin-related lectin-like gene in wheat is a component of the plant defence system. J. Exp. Bot. 62, 5471–5483. 10.1093/jxb/err226 21862481 PMC3223046

[B76] Xie C. Mao X. Huang J. Ding Y. Wu J. Dong S.. (2011). KOBAS 2.0: a web server for annotation and identification of enriched pathways and diseases. Nucleic Acids Res. 39, W316–W322. 10.1093/nar/gkr483 21715386 PMC3125809

[B77] Yerlikaya A. Okur E. Baykal A. Acılan C. Boyacı I. Ulukaya E. (2015). A proteomic analysis of p53-independent induction of apoptosis by bortezomib in 4T1 breast cancer cell line. J. Proteomics 113, 315325. 10.1016/j.jprot.2014.09.010 25305590

[B78] Zuluaga A. Vega-Arreguín J. Fei Z. Matas A. Patev S. Fry W.. (2015). Analysis of the tomato leaf transcriptome during successive hemibiotrophic stages of a compatible interaction with the oomycete pathogen phytophthora infestans. Mol. Plant Pathol. 17, 42–54. 10.1111/mpp.12260 25808779 PMC6638369

